# A targetable LIFR−NF-κB−LCN2 axis controls liver tumorigenesis and vulnerability to ferroptosis

**DOI:** 10.1038/s41467-021-27452-9

**Published:** 2021-12-17

**Authors:** Fan Yao, Yalan Deng, Yang Zhao, Ying Mei, Yilei Zhang, Xiaoguang Liu, Consuelo Martinez, Xiaohua Su, Roberto R. Rosato, Hongqi Teng, Qinglei Hang, Shannon Yap, Dahu Chen, Yumeng Wang, Mei-Ju May Chen, Mutian Zhang, Han Liang, Dong Xie, Xin Chen, Hao Zhu, Jenny C. Chang, M. James You, Yutong Sun, Boyi Gan, Li Ma

**Affiliations:** 1 Hubei Hongshan Laboratory, College of Life Science and Technology, College of Biomedicine and Health, Huazhong Agricultural University, Wuhan, Hubei 430070 China; 2 Department of Experimental Radiation Oncology, The University of Texas MD Anderson Cancer Center, Houston, TX 77030 USA; 3 Houston Methodist Cancer Center, Houston Methodist Hospital, Houston, TX 77030 USA; 4 Department of Bioinformatics and Computational Biology, The University of Texas MD Anderson Cancer Center, Houston, TX 77030 USA; 5 Institute of Biosciences and Technology, Texas A&M University, Houston, TX 77030 USA; 6 CAS Key Laboratory of Nutrition, Metabolism and Food Safety, Shanghai Institute of Nutrition and Health, University of Chinese Academy of Sciences, Chinese Academy of Sciences, Shanghai, 200031 China; 7 Department of Bioengineering and Therapeutic Sciences, University of California San Francisco, San Francisco, CA 94143 USA; 8 Children’s Research Institute, Departments of Pediatrics and Internal Medicine, Center for Regenerative Science and Medicine, University of Texas Southwestern Medical Center, Dallas, TX 75390 USA; 9 Department of Hematopathology, The University of Texas MD Anderson Cancer Center, Houston, TX 77030 USA; 10 Department of Molecular and Cellular Oncology, The University of Texas MD Anderson Cancer Center, Houston, TX 77030 USA; 11 The University of Texas MD Anderson UTHealth Graduate School of Biomedical Sciences, Houston, TX 77030 USA

**Keywords:** Cell death, Liver cancer

## Abstract

The growing knowledge of ferroptosis has suggested the role and therapeutic potential of ferroptosis in cancer, but has not been translated into effective therapy. Liver cancer, primarily hepatocellular carcinoma (HCC), is highly lethal with limited treatment options. LIFR is frequently downregulated in HCC. Here, by studying hepatocyte-specific and inducible Lifr-knockout mice, we show that loss of Lifr promotes liver tumorigenesis and confers resistance to drug-induced ferroptosis. Mechanistically, loss of LIFR activates NF-κB signaling through SHP1, leading to upregulation of the iron-sequestering cytokine LCN2, which depletes iron and renders insensitivity to ferroptosis inducers. Notably, an LCN2-neutralizing antibody enhances the ferroptosis-inducing and anticancer effects of sorafenib on HCC patient-derived xenograft tumors with low LIFR expression and high LCN2 expression. Thus, anti-LCN2 therapy is a promising way to improve liver cancer treatment by targeting ferroptosis.

## Abstract (web summary)

Leukemia inhibitory factor receptor (LIFR) is frequently downregulated in liver cancer. Here the authors show that loss of LIFR promotes liver tumorigenesis and confers resistance to drug-induced ferroptosis through NF-κB-mediated upregulation of iron-sequestering cytokine LCN2.

## Introduction

Liver cancer, arising from genetic and epigenetic alterations, remains a top cause of cancer-related mortality and is one of the most rapidly increasing types of cancer in the United States^1^. Liver cancer subtypes include intrahepatic cholangiocarcinoma, hepatoblastoma, and hepatocellular carcinoma (HCC), which originates from hepatocytes and accounts for the majority of primary liver cancers^2^. While surgical resection and transplantation are recommended for patients with early-stage liver cancer^3,4^, systemic therapies are recommended for advanced HCC^2^. The most common genetic alterations in HCC are mutations in the *TERT* promoter (60%), *TP53* (30%), *CTNNB1* (30%), *ARID1A* (10%), *AXIN1* (10%), *ARID2* (5%), *CCND1* (5–10%), and *VEGFA* (5–10%)^2,5^. Unfortunately, HCC is among the solid tumors with the fewest somatic mutations that can be targeted by molecular therapies^6^. The multi-kinase inhibitor sorafenib, which was the first systemic drug approved by the FDA for first-line treatment of HCC, prolongs patient survival by no more than 3 months^7,8^. To date, most systemic therapies tested in phase 3 trials for advanced HCC have failed to improve on or parallel the efficacy of sorafenib^2^. Recently, clinical trials of nivolumab and pembrolizumab, two immune checkpoint inhibitors targeting PD-1, showed unprecedented responses in some individuals with HCC^9,10^; moreover, atezolizumab plus bevacizumab was the first treatment demonstrating survival benefit for any systemic therapies compared with the standard of care sorafenib in unresectable HCC^11^. However, only a small subset of patients are responders to immunotherapies, and no biomarker to predict response is available in HCC^12^. Thus, identifying the best therapeutic options and predictive biomarkers still represents a major challenge.

As a weak inducer of apoptosis^13^, sorafenib has recently been reported to induce ferroptosis in cancer cell lines^14–16^. Ferroptosis is a type of non-apoptotic cell death characterized by the iron-dependent accumulation of lipid hydroperoxides^17–19^. Thus far, the growing knowledge of ferroptosis regulators and ferroptosis-inducing compounds (most of them have been tested only in cell-line models, which may not always recapitulate therapy response of autochthonous tumors and clinical cancers) have not been translated into clinical benefits, and outstanding questions remain to be addressed. In particular, what genetic or epigenetic alterations in human cancer play a major role in the vulnerability to ferroptosis in vivo? Moreover, it is important to find ways to sensitize therapy-resistant cancers to ferroptosis effectively and safely.

Early work showed that tumor suppressor genes, including *APC*, *GSTP1*, and *CDH1*, are commonly methylated in liver cancer^20,21^. Subsequent studies of HCC revealed additional hypermethylated genes. Some of these genes were hypothesized to be liver tumor suppressors but have not been functionally characterized in HCC in vivo; one of such genes is *LIFR*^22–25^, encoding leukemia inhibitory factor receptor^26^. Complete Lifr-knockout mice die within 24 h of birth and exhibit neuronal, skeletal, metabolic, and placental defects^27^. Although tissue-specific Lifr ablation in the mouse uterine epithelium results in embryo implantation failure^28^, the functions of Lifr in other adult organs, including the liver, remain unknown. In this work, we show that deletion of LIFR promotes liver tumorigenesis and confers resistance to drug-induced ferroptosis through NF-κB-mediated upregulation of iron-sequestering cytokine LCN2.

## Results

### LIFR is downregulated in HCC and loss of Lifr promotes liver cancer

**Fig. 1:**
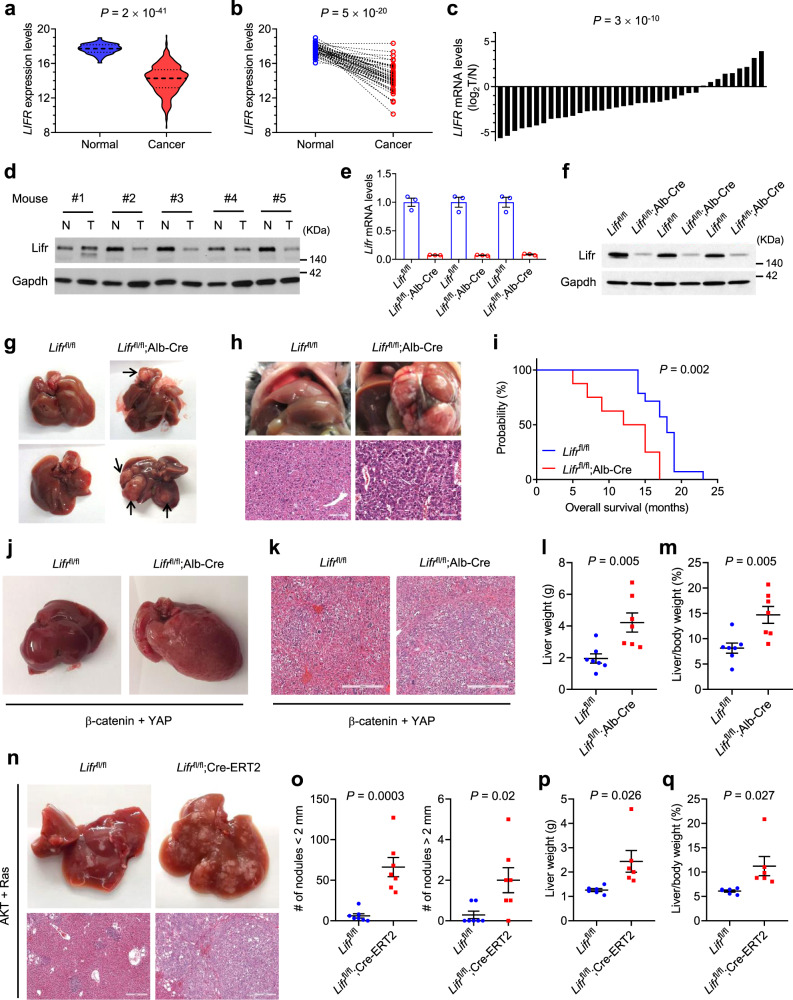
Loss of Lifr promotes liver cancer. **a**, **b** Unpaired (**a**, *n* = 50 patients for normal livers; *n* = 374 patients for liver tumors) and paired (**b**, *n* = 50 patients) comparison of *LIFR* mRNA levels, based on TCGA data. The dashed and dotted lines in **a** are the medians and the quartiles, respectively. Statistical significance was determined by a two-tailed unpaired (**a**) or paired (**b**) *t*-test. **c** qPCR of *LIFR* in human liver tumors (*n* = 37). Statistical significance was determined by a two-tailed unpaired *t*-test. **d** Immunoblotting of Lifr and Gapdh in liver tumors (T) and adjacent normal tissues (N) from DEN-treated C57BL/6 mice. **e**, **f** qPCR (**e**) and immunoblotting (**f**) of Lifr in livers of 3-month-old *Lifr*^fl/fl^ and *Lifr*^fl/fl^;Alb-Cre mice. *n* = 3 samples per mouse; *n* = 3 mice per group. **g** Images of livers in 2-year-old *Lifr*^fl/fl^ and *Lifr*^fl/fl^;Alb-Cre mice. Arrows indicate macroscopic tumors. **h** Images and H&E staining of livers in DEN-treated 7-month-old *Lifr*^fl/fl^ and *Lifr*^fl/fl^;Alb-Cre mice. Scale bars, 100 μm. **i** Kaplan−Meier curves of overall survival of DEN-treated *Lifr*^fl/fl^ (*n* = 14) and *Lifr*^fl/fl^;Alb-Cre (*n* = 8) mice. Statistical significance was determined by a log-rank test. **j**, **k** Images (**j**) and H&E staining (**k**) of livers from *Lifr*^fl/fl^ and *Lifr*^fl/fl^;Alb-Cre mice, 29 days after hydrodynamic injection of plasmids expressing the Sleeping Beauty transposase, β-catenin, and YAP. Scale bars, 300 μm. **l**, **m** Liver weight (**l**) and liver-to-body weight ratio (**m**) of the mice described in **j**. *n* = 7 mice. **n** Gross images and H&E staining of livers from *Lifr*^fl/fl^ and *Lifr*^fl/fl^;Cre-ERT2 mice, 28 days after hydrodynamic injection of plasmids expressing the Sleeping Beauty transposase, myrAKT, and RasV12. From day 7, all mice received 5-day tamoxifen treatment. Scale bars, 300 μm. **o**–**q** Number of liver nodules (*n* = 7 mice; **o**), liver weight (*n* = 6 mice; **p**), and liver-to-body weight ratio (*n* = 6 mice; **q**) of the mice described in **n**. Statistical significance in **l**, **m**, and **o**−**q** was determined by a two-tailed unpaired *t*-test. Error bars are s.e.m. Source data are provided as a Source Data file.

Based on the RNA-sequencing (RNA-seq) data from TCGA^29^ and qPCR analysis of individual cases, *LIFR* mRNA levels were commonly downregulated in HCCs compared with normal tissues (Fig. 1a–c). Consistent with a previous report that 47.9% of human liver tumors showed promoter hypermethylation of the *LIFR* gene^22^, analysis of TCGA data revealed a significant inverse correlation between *LIFR* gene methylation levels and *LIFR* mRNA levels in HCC (Supplementary Fig. 1a), suggesting that DNA methylation may contribute to (but may not be the only cause of) *LIFR* downregulation in HCC. We then analyzed N-nitrosodiethylamine (DEN)-induced mouse liver tumors. At 1 year of age, four of five DEN-treated C57BL/6 mice showed underexpression of Lifr in liver tumors relative to paired normal liver tissues (Fig. 1d). LIFR was also underexpressed in liver cancer cell lines (Supplementary Fig. 1b–d). Compared with MIHA, a non-transformed human hepatocyte cell line^30^, five of six human liver cancer cell lines examined showed much lower levels of LIFR mRNA and protein (Supplementary Fig. 1b, d). Moreover, the comparison of a pair of isogenic mouse liver cell lines revealed that Lifr mRNA and protein levels were much lower in the highly tumorigenic PHR cell line than in the weakly tumorigenic PHM cell line (Supplementary Fig. 1c, d).

To study the function of Lifr, we generated mice with the LoxP-flanked (floxed) *Lifr* allele on a C57BL/6 strain and obtained *Lifr*^flox/flox^ (*Lifr*^fl/fl^) homozygotes from intercrossing between *Lifr*^flox/+^ heterozygotes (Supplementary Fig. 2a, b). We then generated hepatocyte-specific *Lifr* deletion mutants (*Lifr*^fl/fl^;Alb-Cre) by using albumin-Cre mice^31^. As expected, *Lifr*^fl/fl^;Alb-Cre mice showed diminished Lifr mRNA and protein in livers (Fig. 1e, f). To determine liver phenotypes, we followed up one cohort of mice until they reached 2 years of age. At that age, macroscopic liver tumors were observed in four of the 14 *Lifr*^fl/fl^;Alb-Cre mice (tumor numbers: 1, 2, 3, and 4 in those four mice), whereas none of the 12 *Lifr*^fl/fl^ mice showed visible tumors (Fig. 1g). In parallel, we treated *Lifr*^fl/fl^ and *Lifr*^fl/fl^;Alb-Cre mice with a single intraperitoneal injection of 20 mg kg^−1^ DEN at 14 days of age. Seven months later, we euthanized all mice and detected substantial HCC tumor burdens in 4 of the 20 *Lifr*^fl/fl^;Alb-Cre mice, but not in any of the 15 *Lifr*^fl/fl^ controls (Fig. 1h). Moreover, *Lifr*^fl/fl^;Alb-Cre mice had a much higher incidence (7/20) of nuclear atypia than *Lifr*^fl/fl^ mice (1/15) (Supplementary Fig. 2c). We also followed up another cohort of DEN-treated mice throughout their lives. In both groups, macroscopic liver tumors were observed in all mice that died or met euthanasia criteria; however, a pronounced difference in overall survival was observed: whereas *Lifr*^fl/fl^;Alb-Cre mice began to die from liver tumors at 5 months of age, *Lifr*^fl/fl^ mice started to die at 14 months of age (median survival: 13.5 months vs 18 months; *P* = 0.002; Fig. 1i). Taken together, these data suggest that Lifr is a suppressor of spontaneous and carcinogen-induced liver tumors.

Next, by using a method that combines hydrodynamic gene delivery and Sleeping Beauty transposon-mediated somatic integration for long-term oncogene expression in mouse hepatocytes^32^, we introduced β-catenin and YAP, two oncogenes that could cooperate to induce liver tumors with features reminiscent of hepatoblastoma^33^, into *Lifr*^fl/fl^ and *Lifr*^fl/fl^;Alb-Cre mice. We found that loss of Lifr markedly aggravated oncogene-induced hepatoblastoma and increased both liver weight and the liver-to-body weight ratio (Fig. 1j–m). Whereas hepatoblastoma is the most common malignancy of the liver in childhood^34^, HCC is the most common form of liver cancer in adults^2^. Because albumin-Cre is effective at converting floxed alleles in the liver of fetal and neonatal mice^35^, we asked what the consequence of Lifr loss in adult mice is. To address this question, we generated *Lifr*^fl/fl^;Cre-ERT2 mice and performed hydrodynamic tail vein (HDTV) injection with plasmids expressing the Sleeping Beauty transposase, myristoylated AKT (myrAKT), and N-RasV12 at 10 weeks of age, considering that these two oncogenes could cooperate to induce HCC in this transposon system^36,37^, and that the Ras-ERK and AKT-mTOR pathways are frequently activated in human HCC^2,36^. One week after HDTV injection, we treated all mice with tamoxifen for 5 days, and we observed strikingly exacerbated HCC in *Lifr*^fl/fl^;Cre-ERT2 mice compared with *Lifr*^fl/fl^ mice (Fig. 1n–q), suggesting that Lifr is a suppressor of oncogene-induced HCC in adults. Conversely, to determine the overexpression effect of LIFR, we performed hydrodynamic injection of C57BL/6 mice with plasmids expressing the Sleeping Beauty transposase, myrAKT, and RasV12. Three days and 17 days after hydrodynamic transfection, we performed tail injection of control adenovirus or LIFR-expressing adenovirus, finding that adenoviral delivery of LIFR increased LIFR protein levels in liver tissues (Supplementary Fig. 2d) and prolonged survival in mice with oncogene-induced HCC (Supplementary Fig. 2e).

To further investigate LIFR’s effect on liver cells, we used two mouse liver progenitor cell lines, PHM (p53-null; c-Myc-overexpressing) and PHR (p53-null; H-RasV12-overexpressing)^38^. By using a CRISPR-Cas9 approach, we generated Lifr-knockout PHM cells (Supplementary Fig. 3a) and seeded the cells at clonogenic densities. Compared with the control PHM cells, loss of Lifr markedly promoted colony formation (Supplementary Fig. 3b, c). Conversely, overexpression of LIFR in PHM and PHR cell lines inhibited their clonogenic ability (Supplementary Fig. 3d–f). The same effects were observed in human liver cancer cell lines, HepG2 and Mahlavu (Supplementary Fig. 3g–i). Further, we performed the colony formation assay in soft agar, a surrogate assay to gauge oncogenic transformation. Whereas knockdown of LIFR in HepG2 cells promoted colony formation in soft agar (Supplementary Fig. 3g, j), ectopic expression of LIFR inhibited the anchorage-independent growth of the highly tumorigenic PHR cell line (Supplementary Fig. 3d, k). Thus, LIFR inhibits the growth of human and mouse liver cells.

### LIFR confers sensitivity to ferroptosis-inducing drugs

**Fig. 2:**
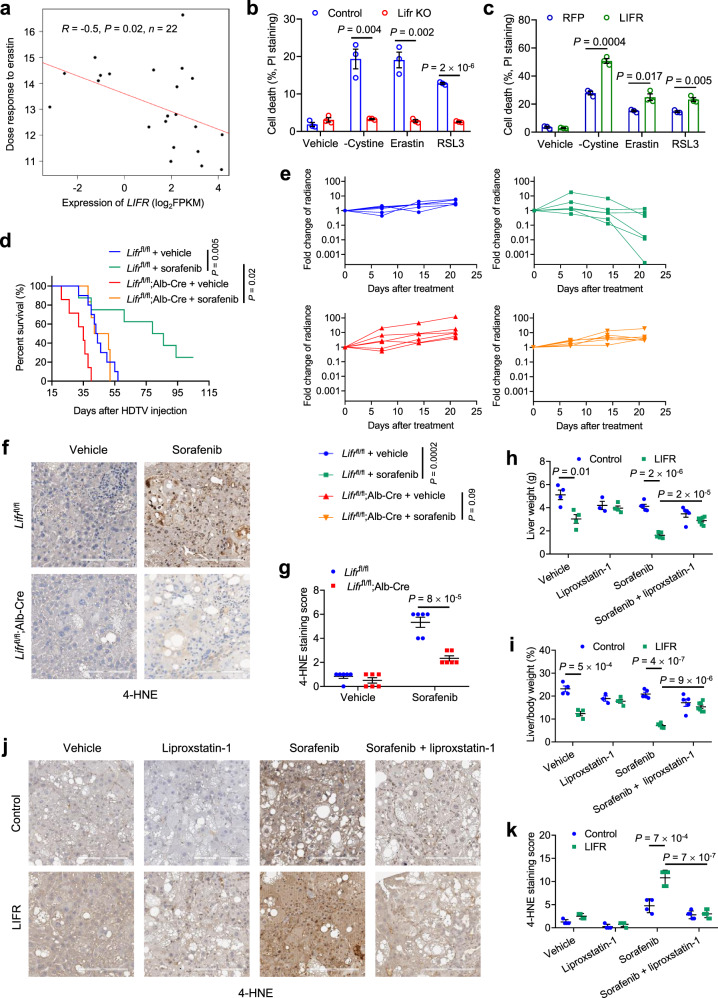
LIFR confers sensitivity to ferroptosis. **a** Correlation between *LIFR* expression and erastin sensitivity, based on the liver cancer cell lines (*n* = 22) from CTRP. Dose responses are normalized area under curve values. The linear relationship was determined by a two-tailed Pearson correlation analysis. **b**, **c** Lifr-knockout (**b**) and LIFR-overexpressing (**c**) PHM cells were treated with DMSO (vehicle), cystine starvation, erastin (10 μM), or RSL3 (0.1 μM) for 24 h. Cell death was measured by PI staining. *n* = 3 wells. **d** Kaplan−Meier curves of overall survival of *Lifr*^fl/fl^ and *Lifr*^fl/fl^;Alb-Cre mice that received sorafenib treatment 7 days after hydrodynamic injection of plasmids expressing the Sleeping Beauty transposase, myrAKT-IRES-luciferase, and RasV12. Sorafenib was administered 6 days a week. Statistical significance was determined by a log-rank test. *n* = 6 mice. **e** Photon flux of *Lifr*^fl/fl^ and *Lifr*^fl/fl^;Alb-Cre mice that received sorafenib treatment 7 days after hydrodynamic injection of plasmids expressing the Sleeping Beauty transposase, myrAKT-IRES-luciferase, and RasV12. Sorafenib was administered 6 days a week. Statistical significance was determined by a two-tailed unpaired *t*-test. *n* = 6 mice. **f**, **g** Immunohistochemical staining (**f**) and quantification (**g**) of 4-HNE in livers of the mice described in **e**. *n* = 6 mice. Scale bars, 200 μm. **h**, **i** Liver weight (**h**) and liver-to-body weight ratio (**i**) of C57BL/6 mice that received control adenovirus or LIFR-expressing adenovirus 3 days and 17 days after hydrodynamic injection of plasmids expressing the Sleeping Beauty transposase, myrAKT, and RasV12. One week after plasmid injection, mice received 30 mg kg^−1^ sorafenib and/or 10 mg kg^−1^ liproxstatin-1, 6 days a week for 4 weeks. *n* = 4, 4, 3, 4, 5, 5, 5, and 7 mice from left to right. **j**, **k** Immunohistochemical staining (**j**) and quantification (**k**) of 4-HNE in livers of the mice described in **h**. *n* = 4, 4, 4, 4, 4, 5, 5, and 7 mice from left to right. Scale bars, 200 μm. Statistical significance in **b**, **c**, **g**–**i**, and **k** was determined by a two-tailed unpaired *t*-test. Error bars are s.e.m. Source data are provided as a Source Data file.

To assess whether LIFR expression is associated with drug response, we used the Cancer Therapeutics Response Portal (CTRP), which enables analysis of the correlations between gene expression and the response to 481 compounds across cancer cell lines^39^. The liver cancer cell line data from the CTRP revealed a significant correlation between *LIFR* expression and the sensitivity to erastin (Fig. 2a), which targets the cystine transporter SLC7A11 to induce ferroptosis^17^. To determine whether LIFR regulates ferroptosis, we challenged liver cells with erastin and another two widely used ferroptosis inducers, RSL3 and cystine withdrawal^17,40^. All three treatments triggered substantial cell death in the control PHM cells; in contrast, Lifr-knockout PHM cells were resistant to ferroptosis induction (Fig. 2b). Conversely, overexpression of LIFR sensitized PHM cells to all three ferroptosis inducers (Fig. 2c).

Recently, sorafenib has been shown to trigger ferroptosis under some conditions^14–16,41–43^. Interestingly, ferroptosis inhibitors, but not inhibitors of apoptosis or necroptosis, can restore HCC cell viability in vitro after sorafenib treatment^42^. This prompted us to examine whether LIFR regulates sorafenib sensitivity. Through HDTV injection, we introduced the Sleeping Beauty transposase, RasV12, and myrAKT-IRES-luciferase into *Lifr*^fl/fl^ and *Lifr*^fl/fl^;Alb-Cre mice. One week later, mice started to receive oral gavage with either the vehicle or sorafenib (30 mg kg^−1^ body weight), once a day, 6 days a week. In the first study cohort, we followed up all mice until the endpoint, finding that in *Lifr*^fl/fl^ mice, sorafenib treatment improved survival substantially (median survival: 43.5 days vs. 82.5 days, Fig. 2d); in contrast, in *Lifr*^fl/fl^;Alb-Cre mice, the survival benefit was modest (median survival: 35 days vs. 46.5 days, Fig. 2d). In the second cohort, we monitored HCC growth by bioluminescent imaging of live animals and observed a significant anti-tumor effect of sorafenib treatment in *Lifr*^fl/fl^ mice (*P* = 0.0002), but not in *Lifr*^fl/fl^;Alb-Cre mice (*P* = 0.09, Fig. 2e). Since ferroptosis is characterized by excessive lipid peroxidation, we performed 4-hydroxy-2-noneal (4-HNE) immunohistochemical analysis^40^ to gauge lipid peroxidation levels, finding that sorafenib treatment increased the 4-HNE level in HCC tumors more prominently in *Lifr*^fl/fl^ mice than in *Lifr*^fl/fl^;Alb-Cre mice (Fig. 2f, g).

Conversely, adenoviral delivery of LIFR not only reduced the growth of AKT- and Ras-induced HCC but also sensitized these tumors to sorafenib treatment, as gauged by the liver weight, the liver-to-body weight ratio, and bioluminescent imaging (Fig. 2h, i and Supplementary Fig. 4); these effects were abrogated by co-treatment with the ferroptosis inhibitor liproxstatin-1^40,44^ (Fig. 2h, i and Supplementary Fig. 4). Moreover, the 4-HNE level in HCC tumors was upregulated by sorafenib treatment (Fig. 2j, k), which could be further elevated by LIFR overexpression and reversed by liproxstatin-1 co-treatment (Fig. 2j, k). Collectively, these data suggest that LIFR inhibits HCC growth and promotes the sensitivity to sorafenib-induced ferroptosis.

### LIFR is a negative regulator of NF-κB signaling and LCN2 in the liver

**Fig. 3:**
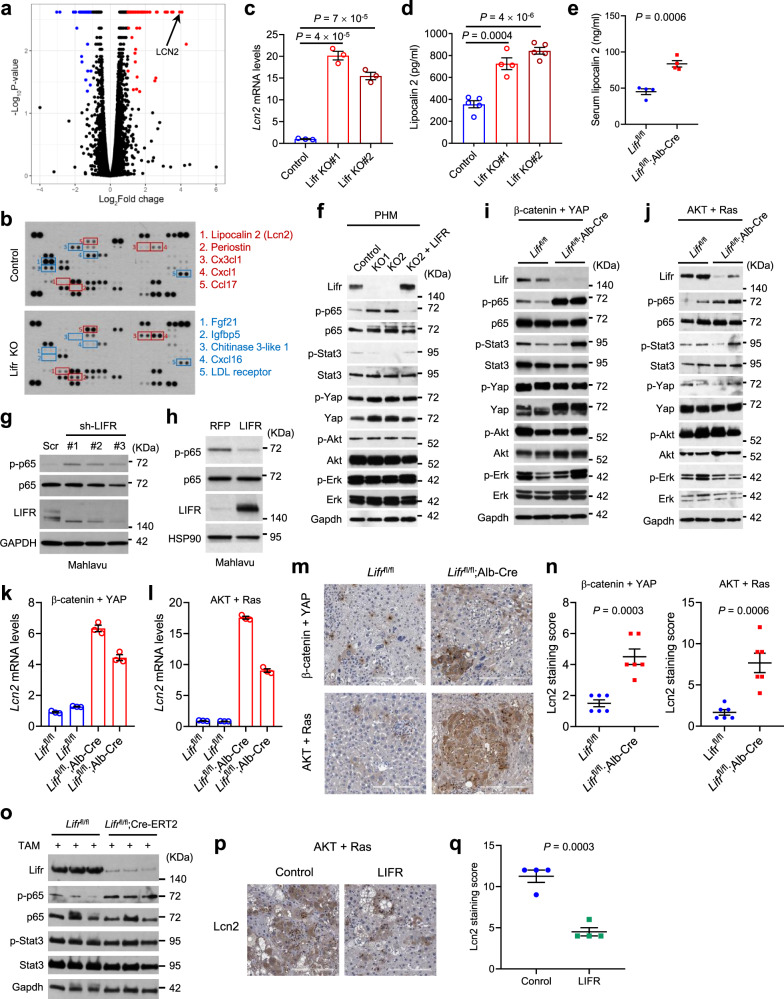
LIFR negatively regulates NF-κB signaling and LCN2 in the liver. **a** Volcano plot of genes upregulated (red) or downregulated (blue) in *Lifr*^fl/fl^;Alb-Cre mice (*n* = 3) relative to *Lifr*^fl/fl^ mice (*n* = 2). Statistical analysis of RNA-seq data was performed using Cuffdiff and *P* values are false discovery rate (FDR)-adjusted. **b** Cytokine arrays of the conditioned medium of Lifr-knockout PHM cells. Boxed: the top five upregulated (red) and downregulated (blue) cytokines. **c** qPCR of *Lcn2* in Lifr-knockout PHM cells. *n* = 3 samples. **d** ELISA of lipocalin 2 in the conditioned medium of Lifr-knockout PHM cells. *n* = 5, 4, and 5 wells from left to right. **e** ELISA of lipocalin 2 in the serum of 3-month-old *Lifr*^fl/fl^ and *Lifr*^fl/fl^;Alb-Cre mice. *n* = 4 mice. **f** Pathway analysis of Lifr-knockout PHM cells with or without LIFR add-back. **g**, **h** Immunoblotting of p-p65, p65, and LIFR in LIFR-knockdown (**g**) and LIFR-overexpressing (**h**) Mahlavu cells. **i**, **j** Pathway analysis of livers of *Lifr*^fl/fl^ and *Lifr*^fl/fl^;Alb-Cre mice that received hydrodynamic injection of plasmids expressing the Sleeping Beauty transposase and oncogenes (**i**: β-catenin + YAP; **j**: myrAKT + RasV12). **k**, **l** qPCR of *Lcn2* in livers described in **i** and **j**, respectively. *n* = 3 samples per mouse; *n* = 2 mice per group. **m**, **n** Immunohistochemical staining (**m**) and quantification (**n**) of Lcn2 in livers described in **i** and **j**, respectively. *n* = 6 mice. **o** Immunoblotting of Lifr, p-p65, p65, p-Stat3, Stat3, and Gapdh in livers of *Lifr*^fl/fl^ and *Lifr*^fl/fl^;Cre-ERT2 mice, 28 days after hydrodynamic injection of plasmids expressing the Sleeping Beauty transposase, myrAKT, and RasV12. From day 7, all mice received 5-day tamoxifen treatment. **p**, **q** Immunohistochemical staining (**p**) and quantification (**q**) of Lcn2 in livers of the mice that received control or LIFR-expressing adenovirus 3 days and 17 days after hydrodynamic injection of plasmids expressing the Sleeping Beauty transposase, myrAKT, and RasV12. Scale bars, 200 μm. *n* = 4 mice. Statistical significance in **c**–**e**, **n**, and **q** was determined by a two-tailed unpaired *t*-test. Error bars are s.e.m. Source data are provided as a Source Data file.

To determine the effect of Lifr loss on the liver transcriptome, we performed RNA-seq analysis of liver tissues from *Lifr*^fl/fl^ and *Lifr*^fl/fl^;Alb-Cre mice. One of the top upregulated genes in the livers of *Lifr*^fl/fl^;Alb-Cre mice, *Lcn2* (encoding lipocalin 2; Fig. 3a and Supplementary Data 1), has been reported to promote mammary tumorigenesis and metastasis^45,46^, and its expression is upregulated in liver cancer^47–50^. Since Lcn2 has cytosolic and secreted forms and can be secreted by neutrophils and hepatocytes^51^, we profiled cytokines secreted by control and Lifr-knockout PHM cells, finding that Lcn2 was one of the top five upregulated cytokines upon Lifr loss (Fig. 3b). We further validated the upregulation of Lcn2 in Lifr-knockout PHM cells by qPCR (Fig. 3c) and ELISA (Fig. 3d), and in the serum of *Lifr*^fl/fl^;Alb-Cre mice by ELISA (Fig. 3e).

LCN2 is a player in iron homeostasis and inflammation^51^. Its expression is induced by cytokines and lipopolysaccharide, and the NF-κB pathway is the main signaling pathway that activates *LCN2* transcription^52,53^. To determine whether LIFR regulates NF-κB signaling, we examined the phosphorylation level of the key transcription factor in the NF-κB pathway, p65 (encoded by *RELA*), because phospho-p65 (at serine 536) is a well-established indicator of NF-κB signaling activity^54,55^. Notably, knockout of Lifr drastically increased p65 phosphorylation in PHM cells, which could be reversed by re-expression of LIFR (Fig. 3f); the same effect was observed in LIFR-knockdown human liver cancer cell lines, Mahlavu and PLC/PRF/5 (Fig. 3g and Supplementary Fig. 5a). Moreover, knockdown of LIFR increased the activity of a luciferase reporter containing either the human *LCN2* promoter or the NF-κB-binding site (Supplementary Fig. 5b). Conversely, overexpression of LIFR in cell lines reduced both p65 phosphorylation (Fig. 3h and Supplementary Fig. 5c) and the activity of a luciferase reporter driven by the human *LCN2* promoter or the NF-κB-binding site (Supplementary Fig. 5d, e).

Previously, LIF or LIFR has been reported to regulate JAK-STAT3, MAPK, PI3K, and Hippo-YAP pathways in a tissue type-dependent manner^56–61^. Similar to Lifr-deficient PHM cells (Fig. 3f), Lifr-knockout mouse livers showed upregulation of both p65 phosphorylation (Fig. 3i, j) as well as mRNA (Fig. 3k, l) and protein (Fig. 3m, n) levels of Lcn2, indicating activation of NF-κB signaling. In contrast, Lifr-deficient livers did not show a substantial decrease in the phosphorylation of Stat3, Yap, Akt, and Erk (Fig. 3i, j). Moreover, tamoxifen-induced loss of Lifr upregulated p65 phosphorylation but did not affect Stat3 phosphorylation in the liver (Fig. 3o). Conversely, Lcn2 was downregulated in LIFR-overexpressing PHM cells (Supplementary Fig. 5f, g); in an oncogene-induced HCC model, adenoviral delivery of LIFR decreased p65 phosphorylation (Supplementary Fig. 5h) and Lcn2 proteins levels (Fig. 3p, q) in liver tissues, and reduced levels of secreted Lcn2 in the serum (Supplementary Fig. 5i, j).

### Loss of LIFR activates NF-κB signaling through SHP1, leading to upregulation of LCN2

**Fig. 4:**
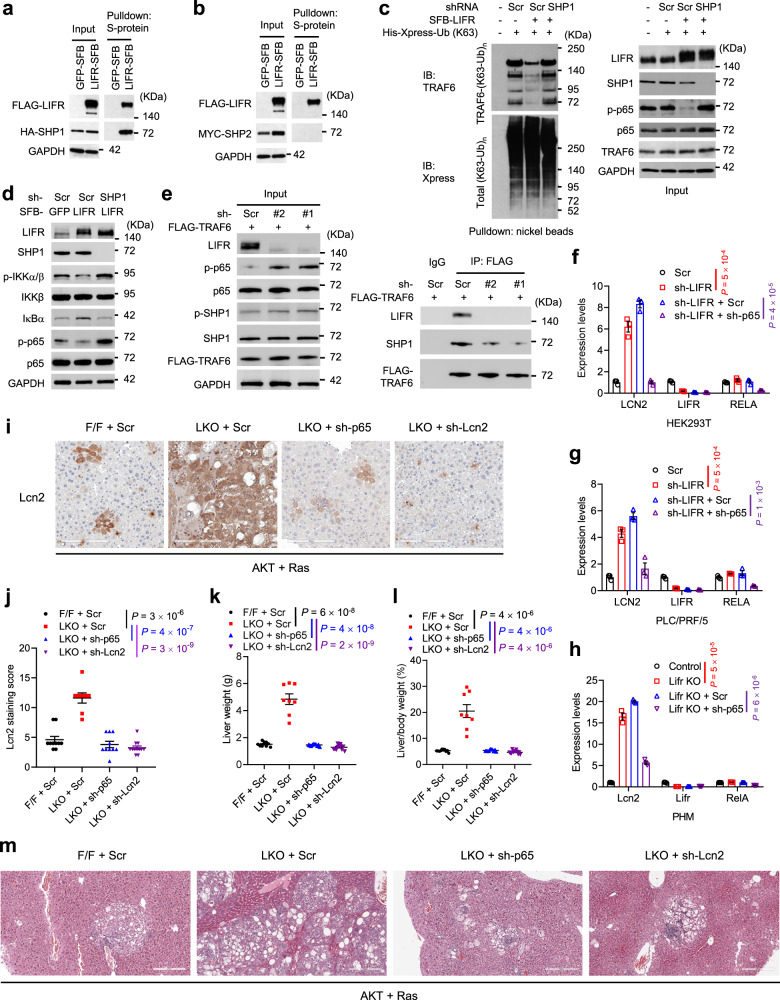
Loss of LIFR activates NF-κB signaling through SHP1, leading to upregulation of LCN2. **a** HEK293T cells were transfected with HA-FLAG-SHP1 and SFB-tagged GFP or LIFR. LIFR-SFB protein was pulled down with S-protein beads, followed by immunoblotting with antibodies against FLAG and HA. **b** HEK293T cells were transfected with MYC-SHP2 and SFB-tagged GFP or LIFR. LIFR-SFB protein was pulled down with S-protein beads, followed by immunoblotting with antibodies against FLAG and MYC. **c** HEK293T SFB-GFP and SFB-LIFR stable cell lines were infected with the scrambled (Scr) or sh-SHP1 lentivirus, followed by transfection with a K63-specific mutant of His-Xpress-ubiquitin (Ub). 48 h later, cells were subjected to pulldown with nickel beads and immunoblotting with antibodies against TRAF6 and Xpress. **d** Control and LIFR-overexpressing PLC/PRF/5 cells were transduced with SHP1 shRNA and immunoblotted with the indicated antibodies. **e** Control (Scr) and LIFR-knockdown HEK293T cells were transfected with FLAG-TRAF6. 48 h later, cells were immunoprecipitated with a FLAG-specific antibody and immunoblotted with antibodies against LIFR, SHP1, and FLAG. **f**, **g** qPCR of *LCN2*, *LIFR*, and *RELA* in HEK293T (**f**) and PLC/PRF/5 (**g**) cells transduced with LIFR shRNA alone or in combination with p65 shRNA. *n* = 3 technical replicates. **h** qPCR of *Lcn2*, *Lifr*, and *RelA* in control and Lifr-knockout PHM cells transduced with the scrambled shRNA (Scr) or p65 shRNA. *n* = 3 technical replicates. **i**, **j** Immunohistochemical staining (**i**) and quantification (**j**) of Lcn2 in livers from *Lifr*^fl/fl^ (F/F) and *Lifr*^fl/fl^;Alb-Cre (LKO) mice, 57 days after hydrodynamic injection of plasmids expressing the Sleeping Beauty transposase, myrAKT, RasV12, and shRNA (sh-p65, sh-Lcn2, or scrambled). *n* = 10, 8, 10, and 12 mice from left to right. Scale bars, 200 μm. **k**, **l** Liver weight (**k**) and liver-to-body weight ratio (**l**) of the mice described in **i** and **j**. *n* = 10, 8, 10, and 12 mice from left to right. **m** H&E staining of livers described in **i** and **j**. Scale bars, 300 μm. Statistical significance in **f**−**h** and **j**−**l** was determined by a two-tailed unpaired *t*-test. Error bars are s.e.m. Source data are provided as a Source Data file.

To understand how LIFR regulates NF-κB signaling, we searched a protein-protein interaction database, BioGRID (https://thebiogrid.org/), for LIFR’s potential interacting proteins. Among all candidate interactors, the phosphatase SHP1 was reported to downregulate p65 phosphorylation in liver cancer cells^62^. Indeed, SHP1, but not SHP2, was pulled down with SFB-tagged LIFR protein by S-protein beads, but not with SFB-tagged GFP protein (Fig. 4a, b). SHP1 has been shown to interact with TRAF6 and inhibit K63-linked ubiquitination of TRAF6, leading to inactivation of NF-κB signaling^63,64^. It is also known that the E3 ligase TRAF6 mediates K63-linked ubiquitination and kinase activation of TAK1, which in turn activates IKK in the NF-κB pathway^65,66^. In our study, overexpression of LIFR reduced both K63-linked ubiquitination of TRAF6 and phosphorylation of p65, which could be rescued by knockdown of SHP1 (Fig. 4c), suggesting that LIFR inhibits TRAF6 ubiquitination and NF-κB signaling through SHP1. Consistently, LIFR overexpression in the PLC/PRF/5 human liver cancer cell line led to downregulation of IKKα/β phosphorylation and upregulation of IκBα protein levels, and these effects were abolished by knockdown of SHP1 (Fig. 4d). Moreover, knockdown of LIFR markedly increased p65 phosphorylation and impaired the interaction between SHP1 and TRAF6, without affecting the tyrosine 564 phosphorylation of SHP1, an indicator of SHP1’s phosphatase activity^67^ (Fig. 4e). Taken together, these data suggest that LIFR facilitates the SHP1-TRAF6 interaction, which in turn inhibits K63-linked ubiquitination of TRAF6 and NF-κB signaling.

To determine whether NF-κB mediates the upregulation of LCN2 caused by loss of LIFR, we used shRNA to knock down p65 in LIFR-depleted HEK293T, PLC/PRF/5, and PHM cell lines. In all three cell lines, depletion of p65 reversed the upregulation of *LCN2* induced by the loss of LIFR (Fig. 4f–h). Furthermore, we co-injected plasmids expressing the Sleeping Beauty transposase, myrAKT, RasV12, and shRNA targeting either p65 or Lcn2 (cloned into a Sleeping Beauty transposon vector with a U6 promoter) into *Lifr*^fl/fl^ and *Lifr*^fl/fl^;Alb-Cre mice. Notably, hepatocyte-specific knockout of Lifr markedly increased Lcn2 expression levels in liver tissues, which was reversed by in vivo knockdown of either p65 or Lcn2 (Fig. 4i, j). These data provide direct evidence that NF-κB is required for the upregulation of LCN2 caused by loss of LIFR. Strikingly, either p65 shRNA or Lcn2 shRNA blocked liver tumorigenesis induced by hepatocyte-specific knockout of Lifr (Fig. 4k–m and Supplementary Fig. 5k), suggesting that loss of Lifr promotes liver cancer development through, at least in part, NF-κB and Lcn2.

In addition, we performed gene set enrichment analysis (GSEA) of the RNA-seq data of liver-specific Lifr-knockout mice, which revealed enrichment of canonical NF-κB signaling activity in the livers of *Lifr*^fl/fl^;Alb-Cre mice compared with *Lifr*^fl/fl^ controls (Supplementary Fig. 5l). Moreover, GSEA of the microarray data (GSE76427) of human HCC samples revealed that the TRAF6-mediated NF-κB pathway activity negatively correlated with *LIFR* expression in HCC tumors (Supplementary Fig. 5m). These data further reinforce the in vivo relevance of inhibition of NF-κB signaling by LIFR in mice and humans.

### LIFR and SHP1 positively regulate ferroptosis while LCN2 negatively regulates ferroptosis

To systematically investigate the role of the LIFR-SHP1-LCN2 axis in ferroptosis, we used two independent shRNAs to knock down LIFR, SHP1, or LCN2 in the HT1080 cell line, a widely used model cell line in the ferroptosis field owing to its sensitivity to erastin and other ferroptosis inducers. We treated cells with erastin, RSL3, FIN56, or FINO2, and performed flow cytometry analysis of 7-AAD (a DNA intercalator and an indicator of loss of cell membrane integrity) and annexin V (a marker of phosphatidylserine accessibility). Since 7-AAD and annexin V indicate cell death but do not serve as definitive markers of ferroptosis, apoptosis, or necroptosis, we used the percentage of 7-AAD and annexin V double-negative cells to gauge cell viability, as described previously^68,69^.

**Fig. 5:**
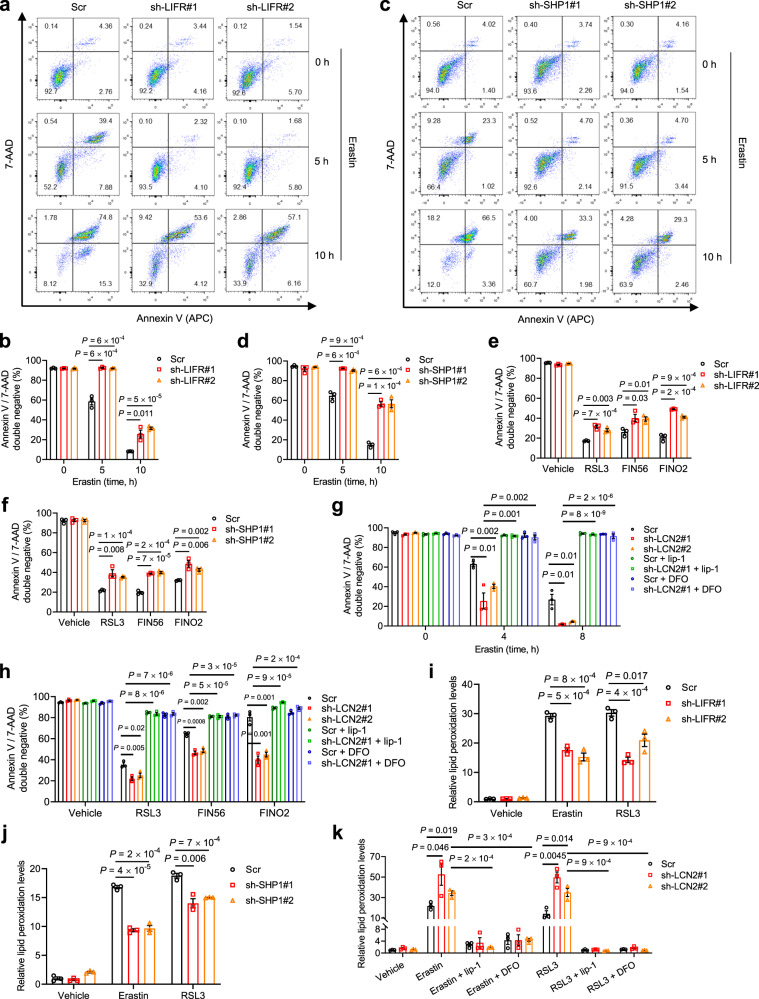
LIFR and SHP1 positively regulate ferroptosis while LCN2 negatively regulates ferroptosis. **a**-**d** LIFR-knockdown (**a**, **b**) or SHP1-knockdown (**c**, **d**) HT1080 cells were treated with 10 μM erastin for 0, 5, or 10 h. **a**, **c**: staining of 7-aminoactinomycin (7-AAD) and annexin V. **b**, **d**: the percentage of annexin V and 7-AAD double-negative population. **e** The percentage of annexin V and 7-AAD double-negative population in LIFR-knockdown HT1080 cells treated with 0.5 μM RSL3 for 12 h, 50 μM FIN56 for 6 h, or 10 μM FINO2 for 24 h. Supplementary Figure 6e shows representative flow cytometry plots. **f** The percentage of annexin V and 7-AAD double-negative population in SHP1-knockdown HT1080 cells treated with 0.5 μM RSL3 for 12 h, 50 μM FIN56 for 6 h, or 10 μM FINO2 for 24 h. Supplementary Figure 6f shows representative flow cytometry plots. **g** The percentage of annexin V and 7-AAD double-negative population in LCN2-knockdown HT1080 cells treated with 10 μM erastin for 0, 4, or 8 h, alone or in combination with liproxstatin-1 (lip-1, 10 μM) or DFO (100 μM). Supplementary Figure 7b shows representative flow cytometry plots. **h** The percentage of annexin V and 7-AAD double-negative population in LCN2-knockdown HT1080 cells treated with 0.5 μM RSL3 for 10 h, 50 μM FIN56 for 3 h, or 10 μM FINO2 for 12 h, alone or in combination with liproxstatin-1 (lip-1, 10 μM) or DFO (100 μM). Supplementary Fig. 7c shows representative flow cytometry plots. **i**, **j** Lipid peroxidation levels in LIFR-knockdown (**i**) and SHP1-knockdown (**j**) HT1080 cells treated with 10 μM erastin for 3 h or 0.5 μM RSL3 for 4 h. **k** Lipid peroxidation levels in LCN2-knockdown HT1080 cells treated with 10 μM erastin for 3 h or 0.5 μM RSL3 for 4 h, alone or in combination with liproxstatin-1 (10 μM) or DFO (100 μM). Lipid peroxidation levels were gauged by C11-BODIPY staining in **i**–**k**. Statistical significance in **b** and **d**–**k** was determined by a two-tailed unpaired *t*-test. Error bars are s.e.m. *n* = 3 samples per group. Source data are provided as a Source Data file.

As expected, knockdown of LIFR or SHP1 in HT1080 cells led to upregulation of *LCN2* (Supplementary Fig. 6a–d). We found that treatment of HT1080 cells with erastin induced cell death over time, and knockdown of either LIFR or SHP1 protected HT1080 cells from cell death at both 5 and 10 h after erastin treatment (Fig. 5a–d). Moreover, LIFR or SHP1 knockdown partially protected against cell death induced by RSL3, FIN56, or FINO2 (Fig. 5e, f and Supplementary Fig. 6e, f). On the other hand, knockdown of LCN2 sensitized HT1080 cells to erastin-induced cell death over time, which could be rescued by co-treatment with the ferroptosis inhibitor liproxstatin-1^40,44^ or the iron chelator deferoxamine (DFO)^51,70^ (Fig. 5g and Supplementary Fig. 7a, b); similar effects were observed in HT1080 cells treated with RSL3, FIN56, or FINO2 (Fig. 5h and Supplementary Fig. 7c). Consistently, in HT1080 cells treated with erastin or RSL3, knockdown of LIFR or SHP1 reduced lipid peroxidation levels, as gauged by C11-BODIPY staining^17,40,71^ (Fig. 5i, j), whereas knockdown of LCN2 increased lipid peroxidation levels, which was reversed by liproxstatin-1 or DFO (Fig. 5k). Taken together, these data further validate that LIFR and SHP1 are positive regulators of ferroptosis, whereas LCN2 is a negative regulator of ferroptosis, which reinforces our results from liver cell lines and tissues.

### LCN2 mediates ferroptosis resistance and is an actionable target for enhancing sorafenib efficacy

The liver stores iron in hepatocytes and is the major organ that controls systemic iron homeostasis^70^. LCN2 sequesters iron by binding to iron-chelating molecules called siderophores, thereby facilitating cellular and systemic hypoferremia^51,72^. This function was initially observed in the anti-bacterial response: bacteria secrete siderophores to acquire iron needed for their survival; however, this is counter-regulated by host-secreted Lcn2, which binds siderophores and blocks iron uptake by bacteria. Consequently, Lcn2-null mice are more sensitive to *E.coli* infection^73^. Moreover, when fed on a high-iron diet, Lcn2-knockout mice showed increased organ damage and elevated serum and liver iron levels^74^. Thus, LCN2 can protect against iron-dependent adverse effects.

**Fig. 6:**
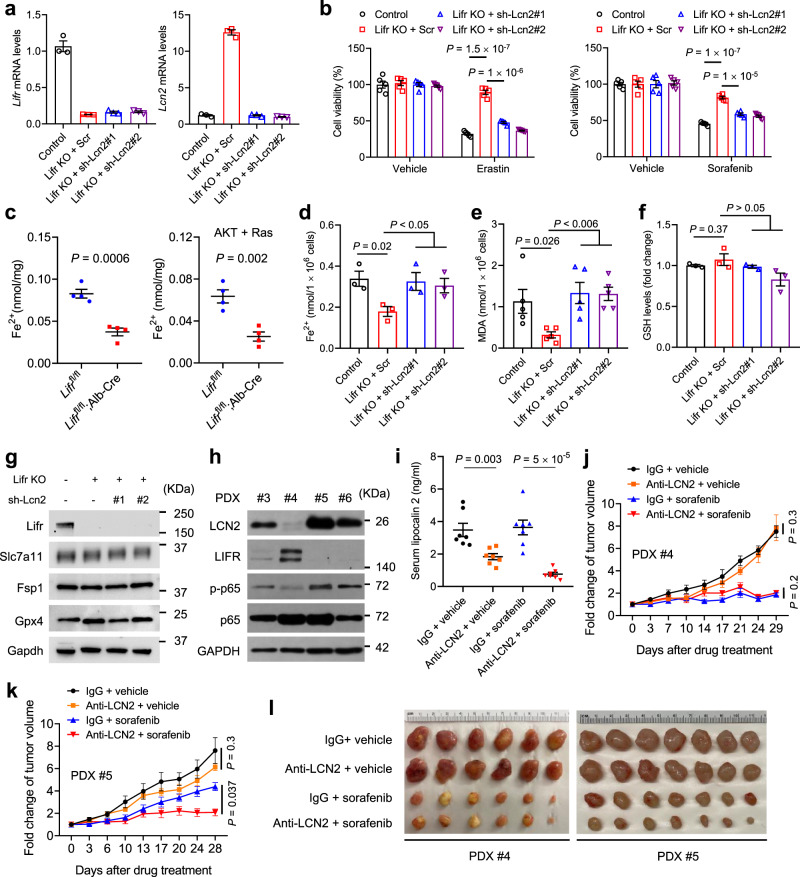
LCN2 mediates ferroptosis resistance and is a therapeutic target for enhancing sorafenib efficacy. **a** qPCR of *Lifr* and *Lcn2* in Lifr-knockout PHM cells transduced with the scrambled (Scr) or Lcn2 shRNA. *n* = 3 samples. **b** Lifr-knockout PHM cells were transduced with Lcn2 shRNA and treated with 10 µM erastin or 20 µM sorafenib. Cell viability was determined by a CCK8 assay. *n* = 5 wells. **c** Fe^2+^ levels in liver tissues of *Lifr*^fl/fl^ and *Lifr*^fl/fl^;Alb-Cre mice in the absence or presence of hydrodynamic injection of plasmids expressing the Sleeping Beauty transposase, myrAKT, and RasV12. *n* = 4 mice. **d** Fe^2+^ levels in control and Lifr-knockout PHM cells transduced with the scrambled (Scr) or Lcn2 shRNA. *n* = 3 wells. **e** Malondialdehyde (MDA) levels in control and Lifr-knockout PHM cells transduced with the scrambled (Scr) or Lcn2 shRNA. *n* = 5 wells. **f** Glutathione (GSH) levels in control and Lifr-knockout PHM cells transduced with the scrambled (Scr) or Lcn2 shRNA. *n* = 3 wells. **g** Immunoblotting of Lifr, Slc7a11, Fsp1, Gpx4, and Gapdh in control and Lifr-knockout PHM cells transduced with the scrambled (Scr) or Lcn2 shRNA. **h** Immunoblotting of LCN2, LIFR, p-p65, p65, and GAPDH in tumors generated from four PDX lines of HCC. **i** ELISA of lipocalin 2 in the serum collected from NSG mice bearing PDX line #5. Mice were treated with anti-LCN2 and sorafenib, alone or in combination. *n* = 7 mice. **j**, **k** Growth curves of tumors in NSG mice bearing PDX line #4 (**j**) or #5 (**k**). When tumors grew to 50–150 mm^3^, mice were treated with 100 μg anti-LCN2 and 30 mg kg^−1^ sorafenib, alone or in combination. The treatments were given 6 days a week for 4 weeks. Statistical significance was determined by a two-way ANOVA. *n* = 6 mice in **j** and *n* = 7 mice in **k**. **l** Endpoint tumor images of the mice described in **j** and **k**. Statistical significance in **b**–**f** and **i** was determined by a two-tailed unpaired *t*-test. Error bars are s.e.m. Source data are provided as a Source Data file.

Based on the Liver Cancer Model Repository (LIMORE)^75^ and Liver Cancer Cell Line (LCCL)^76^ data, *LCN2* expression levels in human liver cancer cell lines correlated with the resistance to erastin and sorafenib (Supplementary Fig. 8a, b). We reasoned that LIFR regulates ferroptosis by downregulating LCN2. Indeed, overexpression of LIFR sensitized PLC/PRF/5 cells to erastin or sorafenib, which could be reversed by co-treatment with liproxstatin-1, DFO, or purified LCN2 protein (Supplementary Fig. 8c), indicating that LIFR sensitizes liver cancer cells to ferroptosis inducers through iron and LCN2. Conversely, knockdown of LIFR in Mahlavu cells and knockout of Lifr in PHM cells upregulated *LCN2* and conferred resistance to erastin and sorafenib, which could be reversed by knockdown of LCN2 (Fig. 6a, b and Supplementary Fig. 8d, e), suggesting that loss of LIFR promotes resistance to ferroptosis-inducing drugs in an LCN2-dependent manner.

Lifr-deficient livers showed downregulation of ferrous iron (Fe^2+^) levels (Fig. 6c), which was consistent with upregulation of Lcn2 (Fig. 3k–n). We hypothesized that depletion of LCN2 increases sensitivity to ferroptosis by upregulating Fe^2+^ and lipid peroxidation levels in cells. Thus, we measured Fe^2+^ levels in the Lifr-knockout PHM mouse liver cell line with or without knockdown of Lcn2. In support of our hypothesis, we found that loss of Lifr decreased cellular levels of Fe^2+^ and lipid peroxidation—as gauged by malondialdehyde (MDA)^71^, a widely used indicator of lipid peroxidation, and both effects were reversed by Lcn2 knockdown (Fig. 6d, e). On the other hand, loss of Lifr or Lcn2 did not change the levels of glutathione (GSH), Slc7a11, Fsp1, and Gpx4 (Fig. 6f, g).

To address whether LCN2-targeting agents can improve the therapeutic efficacy of sorafenib, we established four HCC patient-derived xenograft (PDX) models in NSG (non-obese diabetic; severe combined immunodeficiency; interleukin-2 receptor gamma chain null) mice. Interestingly, three of these four PDX lines (#3−#6) showed low-to-negative expression of LIFR, as well as high levels of LCN2 and phospho-p65 (Fig. 6h). Among all lines, line #5 showed the lowest LIFR level and the highest LCN2 level, while line #4 was the opposite (Fig. 6h).

In preclinical testing, we implanted PDX tumor tissues from lines #4 and #5 into NSG mice and started the treatment when tumors reached 50−100 mm^3^. The mice were assigned to four treatment groups: (1) IgG + vehicle; (2) LCN2 antibody + vehicle; (3) IgG + sorafenib; and (4) LCN2 antibody + sorafenib. Sorafenib was given at 30 mg kg^−1^ body weight by oral gavage once a day until the endpoint, and the LCN2-neutralizing antibody was administered at 100 μg per mouse by intraperitoneal injection once a day until the endpoint, which reduced LCN2 levels in the serum (Fig. 6i). Intriguingly, compared with PDX line #5 (the low-LIFR, high-LCN2 line), PDX line #4 (the high-LIFR, low-LCN2 line) was more sensitive to sorafenib treatment as a single agent (Fig. 6j–l).

**Fig. 7:**
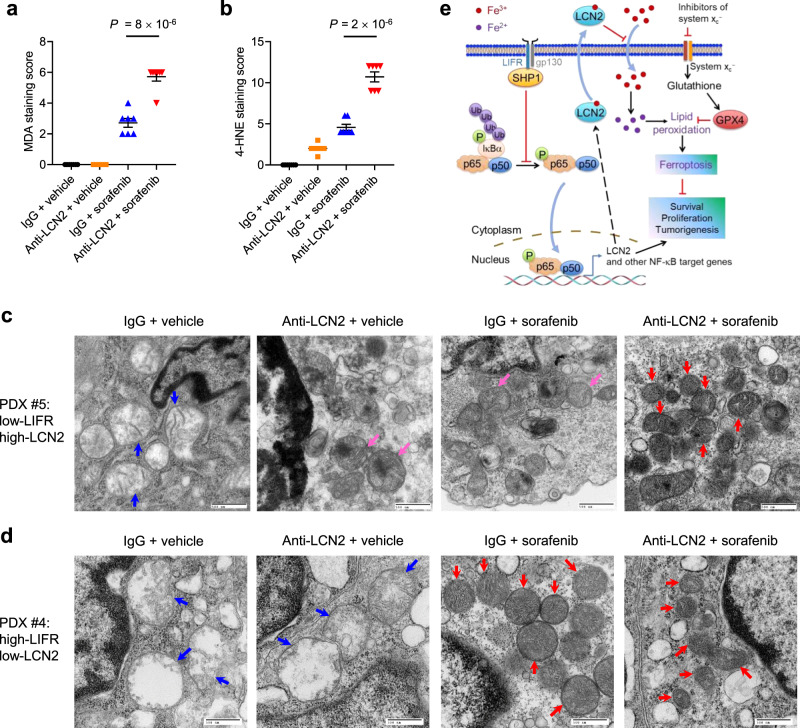
An LCN2-neutralizing antibody enhances the ferroptosis-inducing effect of sorafenib on HCC patient-derived xenograft tumors with low LIFR expression and high LCN2 expression. **a**, **b** Quantification of immunohistochemical staining (IHC, see images in Supplementary Fig. 9a) of MDA (**a**) and 4-HNE (**b**) in tumor tissues from NSG mice bearing PDX line #5. Mice were treated with anti-LCN2 and sorafenib, alone or in combination. *n* = 7 mice. Statistical significance was determined by a one-way ANOVA (to compare the means among three or more groups) and a two-tailed unpaired *t*-test (to compare the means between two groups). Error bars are s.e.m. **c**, **d** Transmission electron microscopy images of tumor tissues from NSG mice bearing PDX line #5 (**c**) or #4 (**d**). Mice were treated with anti-LCN2 and sorafenib, alone or in combination. Blue arrows indicate normal mitochondria. Red arrows indicate shrunken mitochondria with heavily condensed membrane, and pink arrows indicate mitochondria with increased membrane density, but to a lesser extent than those indicated by red arrows. Scale bars, 500 nm. **e** Model for the role of a LIFR−NF-κB−LCN2 axis in liver tumorigenesis and ferroptosis. Source data are provided as a Source Data file.

In mice bearing PDX line #4, treatment with the LCN2-neutralizing antibody did not alter tumor growth either with or without sorafenib co-treatment (Fig. 6j, l). In mice bearing PDX line #5, treatment with the LCN2-neutralizing antibody alone had little effect on tumor growth, whereas the combination treatment achieved a greater anti-tumor effect than sorafenib treatment alone (Fig. 6k, l). We asked whether the combinatory effect on PDX line #5 was associated with ferroptosis. Indeed, immunohistochemical analyses revealed that anti-LCN2 treatment did not affect the levels of the proliferative marker Ki-67 (in viable tumor areas) and the apoptotic marker cleaved caspase-3, but instead exhibited a substantial synergistic effect with sorafenib to elevate the levels of 4-HNE^40^ and MDA^71^ (Fig. 7a, b and Supplementary Fig. 9a–c), two markers of lipid peroxidation. Moreover, electron microscopy analysis revealed that in PDX line #5, tumor cells from the combination treatment group contained shrunken mitochondria with heavily condensed membrane, a morphologic feature of ferroptosis^17,40^; sorafenib or anti-LCN2 treatment alone also increased membrane density of mitochondria, but to a lesser extent than their combination (Fig. 7c). In PDX line #4, either sorafenib treatment alone or the combination treatment induced ferroptosis-associated morphological changes in mitochondria, whereas anti-LCN2 treatment did not alter mitochondria morphology (Fig. 7d). Collectively, these data suggest that the LCN2-neutralizing antibody enhanced the ferroptosis-inducing effect of sorafenib on HCC patient-derived xenograft tumors with low expression of LIFR and high expression of LCN2.

## Discussion

Recent studies have pointed to a tumor-suppressing and metastasis-suppressing role for ferroptosis^77,78^. Both oncoproteins and tumor suppressors, such as YAP, p53, and BAP1, have been shown to control ferroptosis in cancer cells^40,41,79^. However, the growing numbers of ferroptosis regulators are yet to be translated into clinical benefits. Here, by using hepatocyte-specific and inducible Lifr-knockout mice, we demonstrated that Lifr is a bona fide liver tumor suppressor and that its loss confers resistance to ferroptosis-inducing drugs. Mechanistically, LIFR interacts with SHP1 to inhibit NF-κB signaling, leading to repression of the NF-κB target gene *LCN2*, which encodes an iron-sequestering factor (Fig. 7e). Consequently, loss of LIFR not only promotes liver tumorigenesis, but also leads to downregulation of liver iron levels and resistance to ferroptosis (Fig. 7e).

Multiple studies showed a ferroptosis-inducing effect of sorafenib^14–16,41–43^. On the other hand, Conrad and colleagues recently reported (based on in vitro cell culture experiments) that sorafenib did not trigger ferroptosis in a panel of cancer cell lines, and that despite abundant expression of SLC7A11 (the substrate-specific subunit of system xc−), classic system xc− inhibitors (e.g., erastin) could only induce ferroptotic cell death in a subset of tumor cell lines^80^. One compounding factor is cell culture conditions, since Jiang and colleagues reported that sorafenib induced ferroptosis in a manner that is dependent on cell density and Hippo signaling^41^, although the in vivo relevance is unclear. Another possible reason for the insensitivity of cancer cells to sorafenib-induced ferroptosis is that *LIFR* expression is downregulated in a wide range of tumor types, including liver, bladder, breast, colon, kidney, lung, rectum, and thyroid cancers (TCGA data analysis, Supplementary Fig. 10). Thus, future work should determine whether downregulation or loss of LIFR plays a causal role in resistance to sorafenib-induced ferroptosis across multiple types of cancer.

Remarkably, HCC patient-derived xenograft tumors often had low levels of LIFR and high levels of LCN2; when mice bearing these tumors were treated with sorafenib in combination with an LCN2-neutralizing antibody, a much better therapeutic response than sorafenib treatment alone was achieved. Notably, the combination treatment promoted lipid peroxidation and ferroptosis in tumor tissues. On the other hand, PDX tumors with high levels of LIFR and low levels of LCN2, which showed no response to the LCN2-neutralizing antibody, were highly sensitive to sorafenib treatment as a single agent. These findings suggest that high LIFR expression and low LCN2 expression could be used to predict sorafenib responders, and that low LIFR expression and high LCN2 expression could be used to select HCC patients who will likely benefit from the combination therapy with sorafenib and the LCN2-neutralizing antibody. Furthermore, given that radiotherapy and immunotherapy trigger lipid oxidation and ferroptosis via repression of SLC7A11^81^, we envision that the LCN2-neutralizing antibody may have the potential to sensitize tumors to radiation and immune therapies, which warrants future investigation. It should be noted although Lcn2-null mice are more sensitive to bacterial infection and iron overload-induced toxicity, these mice have normal development and growth^73,74^, and thus systemic neutralization of LCN2 is not expected to cause substantial toxicity to normal tissues under physiological conditions. Altogether, this study uncovered an actionable axis governing liver iron homeostasis, tumorigenesis, and vulnerability to ferroptosis, which could pave the way for targeting ferroptosis to improve cancer therapy.

## Methods

### Hepatocyte-specific and inducible Lifr-knockout mouse models

All animal studies were performed in accordance with a protocol approved by the Institutional Animal Care and Use Committee of MD Anderson Cancer Center. Animals were housed at 70 °F−74 °F (set point: 72 °F) with 40–55% humidity (set point: 45%). The light cycle of animal rooms is 12 h of light and 12 h of dark. Through homologous recombination-mediated gene targeting, we generated mice with the LoxP-flanked (floxed) *Lifr* allele (Supplementary Fig. 2a). Briefly, exon 4 was flanked by LoxP sites, and the positive selection marker (the puromycin resistance cassette PuroR) was flanked by FRT sites and inserted into intron 4. The targeting vector was generated using the C57BL/6 J RPCIB-731 BAC library and was transfected into the C57BL/6N Tac ES cell line (Taconic). Homologous recombinant clones were isolated using positive (PuroR) and negative (thymidine kinase—TK) selections, and were injected into C57BL/6 blastocysts to generate chimeric animals. The conditional knockout allele (*Lifr*^flox/+^) was obtained after Flp-mediated removal of the selection marker. *Lifr*^flox/flox^ (*Lifr*^fl/fl^) homozygotes were obtained from intercrossing between *Lifr*^flox/+^ heterozygotes (Supplementary Fig. 2b). Subsequently, hepatocyte-specific *Lifr* deletion mutants (*Lifr*^fl/fl^;Alb-Cre) were generated using albumin-Cre mice (The Jackson Laboratory, stock number: 003574, RRID: IMSR_JAX:003574) in which the Cre transgene is under the control of a hepatocyte-specific albumin promoter^31^. Cre-mediated deletion of exon 4 results in inactivation of the *Lifr* gene by deleting part of the first fibronectin type-III domain and by generating a frameshift and a premature stop codon. In addition, *Lifr*^fl/fl^;Cre-ERT2 mice were generated using the Cre-ERT2 transgenic model (The Jackson Laboratory, stock number: 008085, RRID: IMSR_JAX:008085) in which the Cre-ERT2 fusion gene is under the control of the human ubiquitin C promoter^82^. Primers for PCR genotyping are listed in Supplementary Table 1.

### Carcinogen-induced liver cancer model

One-time intraperitoneal injection of diethylnitrosamine (DEN, 20 mg kg^−1^, dissolved in corn oil and diluted in PBS before injection) was performed in *Lifr*^fl/fl^ and *Lifr*^fl/fl^;Alb-Cre mice at day 14 of age. One cohort of mice were euthanized at 7 months after DEN injection, and the liver tissues were collected and processed for histopathological analysis. Another cohort of mice, used for overall survival analysis, were followed up until death or when they met the institutional criteria for tumor burdens or overall health condition.

### Sleeping beauty transposon-mediated oncogene-induced liver cancer model

Oncogenes (10 μg of each oncogene plasmid along with 2 μg of the SB100 plasmid) were introduced by hydrodynamic injection. The plasmids were diluted in 0.9% sodium chloride solution of the volume equivalent to 10% of body weight, and the injection was finished within 10 seconds through the tail vein. For Lifr loss-of-function studies, 8- to 12-week-old *Lifr*^fl/fl^, *Lifr*^fl/fl^;Alb-Cre, or *Lifr*^fl/fl^;Cre-ERT2 mice were used for hydrodynamic injection, and mice were followed up for overall survival and endpoint analysis. For the *Lifr*^fl/fl^;Cre-ERT2 group and its control (*Lifr*^fl/fl^), mice received 5-day tamoxifen treatment (Sigma-Aldrich, T5648, dissolved in corn oil at a concentration of 20 mg/ml; 100 μl solution was administered by intraperitoneal injection) one week after hydrodynamic injection to induce loss of Lifr. Livers were harvested based on previously reported endpoints^37^ or bioluminescent imaging results (when the myr-AKT-IRES-luciferase construct was used). Bioluminescent imaging of live animals was performed using the IVIS-200 Bioluminescence Imaging System (Perkin Elmer), and each mouse received 100 μl D-luciferin (25 mg ml^−1^ in PBS, Perkin Elmer) immediately before imaging. For Lifr gain-of-function studies, 8-week-old C57BL/6 mice were injected with adenovirus (100 μl of the Adeno-TBG control virus or the Adeno-TBG-LIFR virus, 1 × 10^9^ PFU per mouse) through the tail vein 3 days and 17 days after hydrodynamic transfection with plasmids expressing the Sleeping Beauty transposase, myrAKT, and RasV12. Mice were followed up to determine overall survival. For drug treatment experiments, sorafenib (LC Laboratories, S-8502) stock was dissolved in a solution containing 75% ethanol and Cremophor EL (1:1) at 60 °C, and the stock solution was diluted in water (1:4) before use; liproxstatin-1 (Sigma-Aldrich, SML1414) was dissolved in DMSO and diluted in PBS before use. One week after hydrodynamic transfection of plasmids, mice were treated with sorafenib (30 mg kg^−1^, oral gavage) and/or liproxstatin-1 (10 mg kg^−1^, intraperitoneal injection), once a day, 6 days a week. After 4 weeks of drug treatment, body weight and liver weight were measured, and liver tissues were processed for histopathological analysis and immunohistochemical staining. Living Image^®^ software (Perkin Elmer, for the Xenogen IVIS-200 Imaging System) was used for bioluminescent image analysis and data quantification.

### Patient-derived xenograft (PDX) model

Four HCC PDX lines were from the Houston Methodist Hospital. PDX tumors in cold Dulbecco’s Modified Eagle’s Medium (DMEM) were minced into 1–2 mm^3^ fragments, and each fragment was subcutaneously transplanted into the dorsal flank of 6-week-old male NSG (non-obese diabetic; severe combined immunodeficiency; interleukin-2 receptor gamma chain null) mice. Tumor growth was monitored by bidimensional tumor measurements with a caliper twice a week until the endpoint. The tumor volume was calculated according to the formula volume = 0.5 × length × width^2^. When tumors reached 50-100 mm^3^, mice were randomly assigned to four treatment groups: (1) IgG (100 μg per mouse, R&D Systems, catalog number: MAB006-MTO, clone number: 54447, endotoxin-free, intraperitoneal injection) + vehicle (75% ethanol and Cremophor EL, 1:1, diluted in water, oral gavage); (2) anti-human LCN2 antibody (100 μg per mouse, R&D Systems, catalog number: MAB1757-MTO, clone number: 220310, endotoxin-free, intraperitoneal injection) + vehicle; (3) IgG + sorafenib (30 mg kg^−1^, LC Laboratories, S-8502, oral gavage); and (4) anti-human LCN2 antibody + sorafenib. Mice were euthanized after 4 weeks of drug treatment, and tumors were processed for histopathological analysis, immunohistochemical staining, and transmission electron microscopy.

### Plasmids and shRNA

The pT3-EF1α-AKT-IRES-luciferase plasmid was from Xin Chen’s lab stock. Other Sleeping Beauty transposon-related plasmids, including the Sleeping Beauty transposase (SB100, Addgene number: 34879), pT3-YAP, pT3-β-catenin, pT3-myrAKT, and pT3-N-RasV12, were from Hao Zhu’s lab stock as described previously^37^. Lentiviral human LIFR (Clone ID: PLOHS_100016429) and LentiORF RFP control (Clone ID: OHS5832) plasmids were purchased from Horizon. LIFR-SFB and GFP-SFB plasmids were constructed by Gateway cloning of human LIFR and GFP into the Lenti-SFB (C-terminal tag) destination vector. Human SHP1 and SHP2 were cloned into HA-FLAG-tagged and MYC-tagged destination vectors, respectively, by Gateway cloning. The K63-specific mutant of ubiquitin was cloned into the pcDNA™4/HisMax vector by TA cloning. Human LIFR was also cloned into the Adeno-TBG vector. The Adeno-TBG control virus and Adeno-TBG-LIFR virus were produced at Vector Biolabs. Human LIFR and SHP1 shRNA vectors were purchased from Sigma. shRNA targeting human *LCN2*, human *RELA*, or mouse *RelA* was cloned into the pLKO.1-Blast vector (Addgene number: 26655) with restriction enzymes *Age*I and *EcoR*I. Mouse Lcn2 shRNA vectors were from the Functional Genomics Core at MD Anderson Cancer Center (originally from Horizon). For in vivo knockdown experiments, we inserted the U6 promoter and the shRNA sequence targeting mouse *RelA* or *Lcn2* into the pT4-CMV-GFP Sleeping Beauty transposon vector (Addgene number: 117046) with restriction enzymes *Bgl*II and *Kpn*I. The amounts of the Sleeping Beauty transposase, myrAKT, RasV12, and shRNA used for hydrodynamic transfection are 2, 7, 7, and 7 μg per mouse. shRNA sequence information is provided in Supplementary Table 1.

### Cell culture

The HEK293T cell line was from the Cytogenetics and Cell Authentication Core at MD Anderson Cancer Center. The human liver cell lines (Hep3B, HepG2, Mahlavu, PLC/PRF/5, HA59T, Tong, and Huh-7) were gifts from Mien-Chie Hung (MD Anderson Cancer Center). The immortalized human hepatocyte cell line MIHA was purchased from Albert Einstein College of Medicine. Two immortalized mouse liver progenitor cell lines, PHM (p53-null and c-Myc-overexpressing) and PHR (p53-null and H-RasV12-overexpressing), were from Lars Zender (University of Tübingen) and Wen Xue (University of Massachusetts Medical School), respectively. All cell lines were cultured in DMEM supplemented with 10% fetal bovine serum. Short tandem repeat profiling and mycoplasma tests were done by the Cytogenetics and Cell Authentication Core at MD Anderson Cancer Center.

### Human samples

The human samples were from the Xie laboratory at the Shanghai Institute of Nutrition and Health. All HCC tissues and paired adjacent tissues were collected with written informed consent from Eastern Hepatobiliary Surgery Hospital, Second Military Medical University (Shanghai, China). *LIFR* mRNA levels were determined by qPCR. The collection and use of human samples were approved by the Ethics Committee of Shanghai Institutes for Biological Sciences, Chinese Academy of Sciences (Shanghai, China) following the Declaration of Helsinki ethical guidelines.

### Immunoblotting

Cultured cells or homogenized mouse tissues were lysed in RIPA lysis buffer (Millipore) containing protease inhibitors and phosphatase inhibitors (GenDEPOT). Proteins were resolved on 4–20% precast gradient gels (Bio-Rad) and transferred to a nitrocellulose membrane. After transfer, membranes were blocked with 5% non-fat milk in Tris-buffered saline with 0.05% Tween-20 (TBST) and incubated with the primary antibody at 4 °C overnight, followed by incubation with the secondary antibody conjugated with horseradish peroxidase (HRP). The bands were visualized with enhanced chemiluminescence substrate (Pierce). Primary antibodies used are as follows: antibodies against LIFR (1:2,000, Proteintech, 22779-1-AP, RRID: AB_2879165), GAPDH (1:1,000, ThermoFisher Scientific, MA5-15738, RRID: AB_10977387), HSP90 (1:5,000, BD Biosciences, 610419, RRID: AB_397799), FLAG (1:20,000, Sigma-Aldrich, F7425, RRID: AB_439687), p-p65 (1:500, Cell Signaling Technology, 3033, RRID: AB_331284), p65 (1:1000, Cell Signaling Technology, 6956, RRID: AB_10828935), p-STAT3 (1:300, Cell Signaling Technology, 9145, RRID: AB_2491009), STAT3 (1:1,000, Cell Signaling Technology, 9139, RRID: AB_331757), p-YAP (1:500, Cell Signaling Technology, 4911, RRID: AB_2218913), YAP (1:2000, Cell Signaling Technology, 14074, RRID: AB_2650491), p-AKT (1:500, Santa Cruz Biotechnology, sc-7985, RRID: AB_667741), AKT (1:1,000, Cell Signaling Technology, 2920, RRID: AB_1147620), p-ERK (1:1,000, Cell Signaling Technology, 9101, RRID: AB_331646), ERK (1:1,000, Cell Signaling Technology, 4696, RRID: AB_390780), TRAF6 (1:500, Cell Signaling Technology, 8028, RRID: AB_10858223), SHP1 (1:500, Cell Signaling Technology, 3759, RRID: AB_2173694), SHP2 (1:1,000, Cell Signaling Technology, 3397, RRID: AB_2174959), Xpress (1:2,000, ThermoFisher Scientific, R910-25, RRID: AB_2556552), LCN2 (1:500, R&D Systems, AF1757, RRID: AB_354974), GPX4 (1:1000, R&D Systems, MAB5457, RRID: AB_2232542), FSP1 (1:1000, Proteintech, 20886-1-AP, RRID: AB_2878756), SLC7A11 (1:1000, Cell Signaling Technology, 98051, RRID:AB_2800296), phospho-IKKα/β (Ser176/180) (1:300, Cell signaling technology, 2697s, RRID: AB_2079382), IKKβ (1:1000, Cell signaling technology, 8943s, RRID: AB_11024092), and IκBα (1:1000, Cell signaling technology, 4812s, RRID: AB_10694416). Uncropped blots are provided as a Source Data file.

### Protein pulldown assay

HEK293T cells were transfected with SHP1 or SHP2 along with SFB-tagged GFP or LIFR. 48 h after transfection, cells were lysed in CHAPS buffer (120 mM NaCl, 20 mM NaF, 1 mM EDTA, 25 mM Tris-HCl, pH 7.5, 0.33% CHAPS) containing protease and phosphatase inhibitors (GenDEPOT). For pulldown of SFB-tagged proteins, cell extracts were incubated with S-protein beads (Millipore, 69704) at 4 °C for 2 h, and then the beads were washed with lysis buffer 5 times, followed by Western blot analysis with the indicated antibodies.

### Co-immunoprecipitation

Control and LIFR-knockdown HEK293T cells were transfected with FLAG-TRAF6. 48 h after transfection, cells were lysed in CHAPS buffer (120 mM NaCl, 20 mM NaF, 1 mM EDTA, 25 mM Tris-HCl, pH 7.5, 0.33% CHAPS) containing protease and phosphatase inhibitors. For immunoprecipitation of FLAG-tagged TRAF6 and its interacting proteins, cell extracts were pre-cleared with TrueBlot Agarose (Rockland, TrueBlot^®^ 00-881-25) at 4 °C for 30 min, and were then incubated with an anti-FLAG antibody (Sigma, F1804) at 4 °C for 2 h, followed by incubation with TrueBlot Agarose for additional 1 h. After that, the agarose was washed with lysis buffer for 5 times. Western blot analysis was performed with the indicated antibodies to confirm the interaction. FLAG-tagged TRAF6 was detected by HRP-conjugated TrueBlot^®^ Secondary Antibodies (18-8816-33).

### Tissue processing, histology, and immunohistochemistry (IHC)

After euthanasia, some tissues were frozen for later RNA and protein extraction, and other tissues were fixed in 10% neutral-buffered formalin (ThermoFisher Scientific) overnight, washed with PBS, transferred to 70% ethanol, embedded in paraffin, sectioned (5 μm thick), and stained with hematoxylin and eosin (H&E). Histopathological review was performed on all tissue sections by a pathologist (M. James You). For IHC staining, slides were deparaffinized in xylene and degraded alcohols. Heat-induced epitope retrieval was performed using a 2100-Retriever. Slides were rinsed with PBS, and a hydrophobic barrier was created around the tissue using a hydrophobic barrier pen (Vector Laboratories, H-4000-2). Then, slides were placed in an incubating chamber with blocking solution (Vector Laboratories, SP-6000) for 10 min and rinsed with PBS, followed by incubation with 20% horse serum (Vector Laboratories, PK-7200) for 20 min. Next, slides were incubated with the primary antibody at 4 °C and rinsed with PBS, followed by incubation with a biotinylated universal secondary antibody (Vector Laboratories, PK-7200) or goat IgG HRP-conjugated antibody (R&D systems, HAF017, RRID: AB_562588) for 30 min. After PBS washing, slides were incubated with the avidin-biotin detection complex (ABC; Vector Laboratories, SK-4100) for 30 min and were then developed with 3,3′-diaminobenzidine (DAB) solution (Vector Laboratories, SK-4100). Counterstaining was performed using Hematoxylin QS (Vector Laboratories, H-3404). Primary antibodies used for IHC are as follows: antibodies against 4-hydroxy-2-noneal (4-HNE, 1:400, Abcam, ab46545, RRID: AB_722490), malondialdehyde (MDA, 1:400, AdipoGen, JAI-MMD-030N), Ki-67 (1:200, Cell Signaling Technology, 9027, RRID: AB_2636984), cleaved caspase 3 (1:200, Cell Signaling Technology, 9661, RRID: AB_2341188), and Lcn2 (1:250, R&D Systems, AF1857, RRID: AB_355022). Immunohistochemical staining was semiquantitatively analyzed using the immunoreactive score (IRS) system. The percentage of positive cells was scored on a scale of 0−4: 0 if 0% of tumor cells were positive, 1 if 1−10% were positive, 2 if 11%-50% were positive, 3 if 51−80% were positive, and 4 if 81−100% were positive. The staining intensity was scored on a scale of 0−3 (3 is the strongest). Final IRS score = (score of the staining intensity) × (score of the percentage of positive cells).

### CRISPR-Cas9-mediated gene editing

CRISPR-Cas9-mediated Lifr-knockout constructs were purchased from Santa Cruz Biotechnology (sc-421433). PHM cells were transfected with the Lifr-knockout constructs. 48 h after transfection, the cells were seeded in 96-well plates for single colony isolation. Lifr-null clones were confirmed by Western blot analysis.

### Viral transduction

HEK293T cells were co-transfected with the viral vector and packaging plasmids (psPAX2 and pMD2.G). Two days after transfection, viral supernatant was harvested, filtered through a 0.45 μm filter, and added to target cells. The infected cells were selected with 1 μg ml^−1^ puromycin, 5 μg ml^−1^ blasticidin, or 300 μg ml^−1^ hygromycin B.

### Iron measurement

Mouse liver tissues were harvested and liver iron levels were determined using the Iron Assay Kit (Abcam, ab83366), according to the manufacturer’s protocol. The results were normalized to the tissue weight.

### MDA and GSH measurements

1 × 10^6^ cells were harvested and MDA levels were determined using the Lipid Peroxidation (MDA) Assay Kit (Sigma-Aldrich, MAK085-1KT), according to the manufacturer’s protocol. 1 × 10^5^ cells were harvested and GSH levels were determined using the GSH-Glo™ Glutathione Assay Kit (Promega, V6911), according to the manufacturer’s protocol.

### Flow cytometry

For 7-AAD and annexin V staining, cells were harvested and the pellets were washed twice with cold cell staining buffer (2% FBS in PBS). 1 × 10^6^ cells were resuspended in 100 μl Annexin V Binding Buffer with 5 μl APC Annexin V and 5 μl 7-AAD Viability Staining Solution (BioLegend, 640930). Cells were gently vortexed and then incubated at room temperature (25 °C) in the dark for 15 min. 400 μl of Annexin V Binding Buffer was added to each tube before flow cytometry analysis. For lipid peroxidation measurement, cells were incubated in the culture medium containing 2 μM BODIPY 581/591 C11 (Lipid Peroxidation Sensor) (ThermoFisher, D3861) at 37 °C for 30 min. After staining, cells were washed and resuspended in fresh Cell Staining Buffer (2% FBS in PBS). Cells were processed on an Invitrogen Attune NxT Acoustic Focusing Cytometer and analyzed by FlowJo software. A representative gating strategy for flow cytometry analysis is shown in Supplementary Fig. 11.

### RNA isolation and qPCR

Total RNA was harvested using TRIzol reagent (Invitrogen) and then isolated using the RNeasy Mini Kit (Qiagen) according to the manufacturer’s protocol. Total RNA was reverse transcribed with an iScript complementary DNA (cDNA) Synthesis Kit (Bio-Rad). The resulting cDNA was used for real-time PCR using the iTaq Universal SYBR Green Kit (Bio-Rad). β-actin or Gapdh was used as an internal control. Real-time PCR and data collection were performed on a CFX96 instrument (Bio-Rad). Primers for qPCR are listed in Supplementary Table 1.

### Luciferase reporter assay

Human *LCN2* promoter^83^ (Addgene number: 28225) and NF-κB activity^84^ (Addgene number: 106979) reporter constructs were purchased from Addgene. The firefly luciferase reporter containing the human *LCN2* promoter was co-transfected into HEK293T cells along with a Renilla luciferase vector (for normalization) and the indicated plasmid (control vector or LIFR). The firefly luciferase reporter containing the NF-κB binding site and an internal Renilla luciferase sequence (for normalization) was co-transfected into HEK293T cells along with the indicated plasmid (control vector or LIFR). Two days after transfection, firefly and Renilla luciferase activities were measured using a Dual-Luciferase Reporter Assay (Promega, E1910) on a microplate reader according to the manufacturer’s protocol. Firefly luciferase activity was normalized to Renilla luciferase activity.

### Transmission electron microscopy

Samples were fixed with a solution containing 3% glutaraldehyde and 2% paraformaldehyde in 0.1 M cacodylate buffer (pH 7.3), then washed in 0.1 M sodium cacodylate buffer, treated with 0.1% Millipore-filtered cacodylate buffered tannic acid, postfixed with 1% buffered osmium, and stained en bloc with 1% Millipore-filtered uranyl acetate. The samples were dehydrated in increasing concentrations of ethanol, infiltrated, and embedded in LX-112 medium. The samples were polymerized in a 60 °C oven for approximately 3 days. Ultrathin sections were cut using a Leica Ultracut microtome (Leica, Deerfield, IL), stained with uranyl acetate and lead citrate in a Leica EM Stainer, and examined using a JEM 1010 transmission electron microscope (JEOL, USA, Inc., Peabody, MA) at an accelerating voltage of 80 kV. Digital images were obtained using the AMT Imaging System (Advanced Microscopy Techniques Corp, Danvers, MA) at MD Anderson’s High-Resolution Electron Microscopy Facility.

### RNA-seq analysis

Duplicate RNA samples from liver tissues of 3-month-old *Lifr*^fl/fl^ (*n* = 2 mice) and *Lifr*^fl/fl^;Alb-Cre (*n* = 3 mice) animals were subjected to mRNA sequencing at MD Anderson’s Advanced Technology Genomics Core. The quality of fastq files was determined by Fastqc (V0.11.5, https://www.bioinformatics.babraham.ac.uk/projects/fastqc/). The pair-ended reads were mapped to the mouse mm10 (February 2009, UCSC) genome using Tophat (V2.1.1, https://github.com/infphilo/tophat); only uniquely mapped reads were extracted using SAMtools (V1.5, http://samtools.sourceforge.net/) as inputs for differentially expressed gene analysis. The results from RSeQC (V2.6.4, http://rseqc.sourceforge.net/) indicated high data quality. Cufflinks (V2.2.1, http://cole-trapnell-lab.github.io/cufflinks) was used to assemble the transcriptome using the RefSeq (September 7, 2015) annotation file and to quantitate the gene expression level with fragments per kb of transcript per million mapped reads (FPKM). Differentially expressed genes (DEGs) were identified using Cuffdiff (V2.2.1, http://cole-trapnell-lab.github.io/cufflinks) with the corrected *P* value < 0.05 and |log_2_ fold change | > 1.5. Principle Component Analysis of all DEGs among all samples was performed using Bioconductor package DESeq2 (http://bioconductor.org/packages/release/bioc/html/DESeq2.html).

### Cytokine array and enzyme-linked immunosorbent assay (ELISA)

The conditioned medium from control, Lifr-knockout, and LIFR-expressing PHM cells were collected and centrifuged at 2,500×*g* for 5 min. The supernatant was collected for further use. Whole blood was collected from the hearts of mice immediately after euthanasia, and placed at room temperature for 30 min, followed by centrifugation at 1,000×*g* for 15 min. The supernatant was collected for further use. The cytokine array assay (Mouse XL Cytokine Array Kit, R&D Systems, ARY028) and ELISA kits (Mouse Lcn2 Simplestep ELISA Kit, Abcam, ab199083; Human Lipocalin-2 ELISA Kit, Abcam, ab119600) were performed according to the manufacturer’s protocols.

### In vitro clonogenic assay

The indicated human or mouse liver cell lines were seeded in 6-well or 12-well tissue culture plates at single-cell densities. After colonies formed, cells were fixed and stained with crystal violet (0.05% w/v in formalin). The dye from stained cells was dissolved in 10% acetic acid and the absorbance was measured at 570 nm.

### Soft agar colony formation assay

Human or mouse liver cells were mixed with 0.3% soft agar in culture medium and seeded in 6-well plates (2,000 cells per well) with a bottom layer of 0.6% soft agar in DMEM. After 3 weeks, colonies were photographed and counted.

### Cell death and viability assays

To measure cell death, we seeded cells in a 12-well plate one day before treatment. After treatment with the indicated drugs, cells were trypsinized and collected in a 1.5 ml tube, washed with PBS, and stained with 2 μg ml^−1^ propidium iodide (PI; Roche) in PBS. Dead cells (PI-positive cells) were counted using a BD Accuri C6 flow cytometer (BD Biosciences). To measure cell viability, we seeded 3,000−5,000 cells per well in a 96-well plate one day before treatment. After treatment with the indicated drugs, the medium in each well was replaced with fresh medium containing the Cell Counting Kit-8 (CCK8) reagent (Sigma-Aldrich, 96992). After incubation at 37 °C for 1 h, the plate was analyzed using a FLUOstar Omega microplate reader (BMG Labtech), and the absorbance was measured at 540 nm.

### TCGA and computational data analysis

To compare *LIFR* mRNA levels between normal and tumor tissues, we used TCGA liver cancer RNA-seq data downloaded from the UCSC Xena browser (https://xenabrowser.net/). The gene expression values are expressed as log_2_(FPKM-UQ+1) (https://docs.gdc.cancer.gov/Encyclopedia/pages/HTSeq-FPKM-UQ/), and a paired or unpaired *t*-test was performed to compare two groups. The correlation between mRNAs levels (log_2_RSEM) and methylation levels of *LIFR* in TCGA HCC patients was analyzed by the Pearson correlation test, and the RNA-seq data (RSEM) and methylation data were downloaded from cBioPortal (https://www.cbioportal.org/). To analyze the correlations between gene expression and drug response across cancer cell lines, we used the Cancer Therapeutics Response Portal (CTRP) (http://portals.broadinstitute.org/ctrp.v2.1/), which provides correlation coefficients for gene expression levels and dose responses—expressed as normalized area under curve (AUC) values—for 860 cancer cell lines treated with 481 compounds. The AUC values of CTRP cell lines were downloaded from https://github.com/remontoire-pac/ctrp-reference/tree/master/auc. The gene expression values of CTRP cell lines were downloaded from the Cancer Cell Line Encyclopedia (CCLE) data portal (https://portals.broadinstitute.org/ccle/data). For correlation analyses of *LCN2* expression in liver cancer cell lines with the sensitivity to erastin and sorafenib, we used the Liver Cancer Model Repository (LIMORE) dataset^75^ (https://www.picb.ac.cn/limore/; dose responses are expressed as Emax values) consisting of 81 human liver cancer cell lines and the Liver Cancer Cell Line (LCCL) dataset^76^ (https://lccl.zucmanlab.com/hcc/home; dose responses are expressed as AUC values) consisting of 34 human liver cancer cell lines. Linear relationships between gene expression and drug response were determined by the Pearson correlation analysis. For pan-cancer analysis of *LIFR* expression in normal tissues and tumor tissues, we used the TIMER database (https://cistrome.shinyapps.io/timer/). Gene set enrichment analysis was performed using GSEA (http://www.gsea-msigdb.org/gsea/index.jsp, 4.0.0).

### Statistics and reproducibility

Except for the animal studies (one time), RNA-seq (one time), and cytokine array (one time), each experiment was repeated at least three times with similar results. For qPCR assays, we used *n* = 3 technical replicates per sample, and a representative set from three independent experiments is shown. For all other experiments, we used biological replicates. The statistical analysis for each plot was described in figure legends. Unless otherwise noted, data are presented as mean ± s.e.m, and Student’s *t*-test (two-tailed) was used to compare two groups of independent samples. The data analyzed by the *t*-test meet normal distribution; we used an F-test to compare variances, and the variances are not significantly different. Therefore, when using an unpaired *t*-test, we assumed equal variance, and no data points were excluded from the analysis. *P* < 0.05 was considered statistically significant.

### Reporting summary

Further information on research design is available in the Nature Research Reporting Summary linked to this article.

## Supplementary information

Supplementary Information

Description of Additional Supplementary Files

Supplementary Data 1

Reporting Summary

## Data Availability

The source data that support the findings of this study are available. The RNA-seq raw data have been deposited at the Gene Expression Omnibus (GEO) under the accession number GSE177042. Source data are provided with this paper.

## Source data

Source Data
