# Supplementary material for: A targetable LIFR−NF-κB−LCN2 axis controls liver tumorigenesis and vulnerability to ferroptosis
Source: Nat Commun. 2021 Dec 17;12:7333. doi: 10.1038/s41467-021-27452-9 (PMC8683481; doi:10.1038/s41467-021-27452-9)
Supplement: Supplementary file 1 — Supplementary Information [file 41467_2021_27452_MOESM1_ESM.pdf]

Supplementary Information

A targetable LIFR−NF-κB−LCN2 axis controls liver tumorigenesis and vulnerability to ferroptosis

Fan Yao, Yalan Deng, Yang Zhao, Ying Mei, Yilei Zhang, Xiaoguang Liu, Consuelo Martinez, Xiaohua Su, Roberto R. Rosato, Hongqi Teng, Qinglei Hang, Shannon Yap, Dahu Chen, Yumeng Wang, Mei-Ju May Chen, Mutian Zhang, Han Liang, Dong Xie, Xin Chen, Hao Zhu, Jenny C. Chang, M. James You, Yutong Sun, Boyi Gan, Li Ma

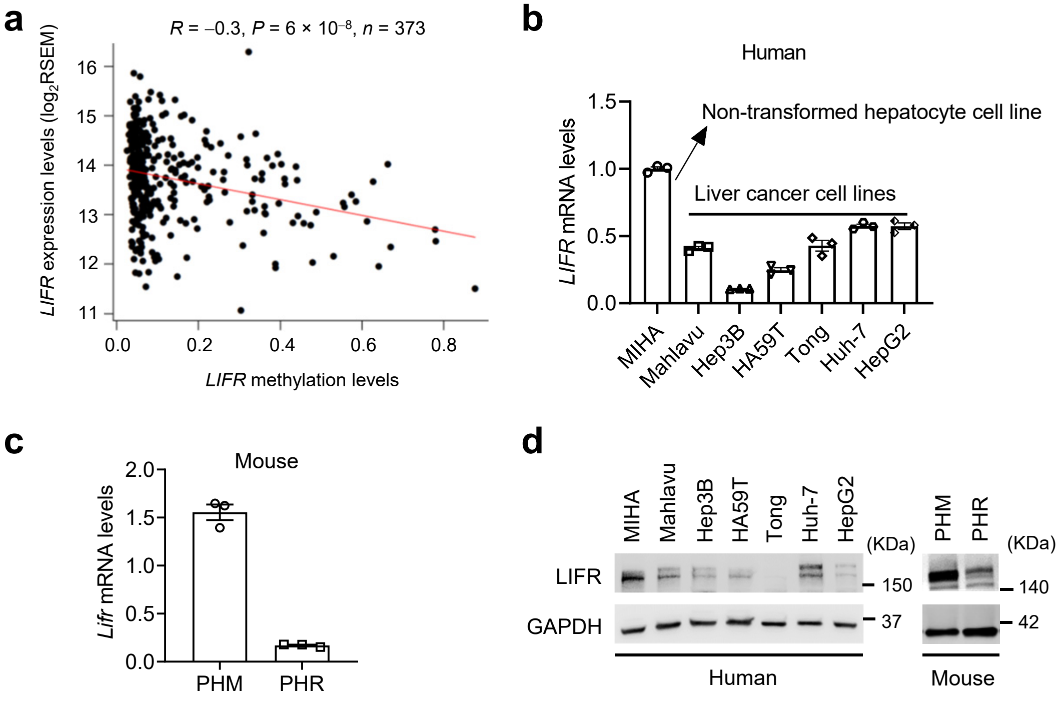

Supplementary Fig. 1. LIFR is downregulated in liver cancer cells.

(**a**) Correlation between mRNA levels (Log_2RSEM) and methylation levels of *LIFR* in HCC, based on TCGA data analysis. Statistical significance was determined by a two-tailed Pearson correlation test.

(**b, c**) qPCR of *LIFR* in human (**b**) and mouse (**c**) liver cell lines. $n = 3$ samples per cell line. Error bars are s.e.m. The PHM cell line is weakly tumorigenic and the PHR cell line is highly tumorigenic.

(**d**) Immunoblotting of LIFR and GAPDH in human and mouse liver cell lines.

Source data are provided as a Source Data file.

Supplementary Fig. 2

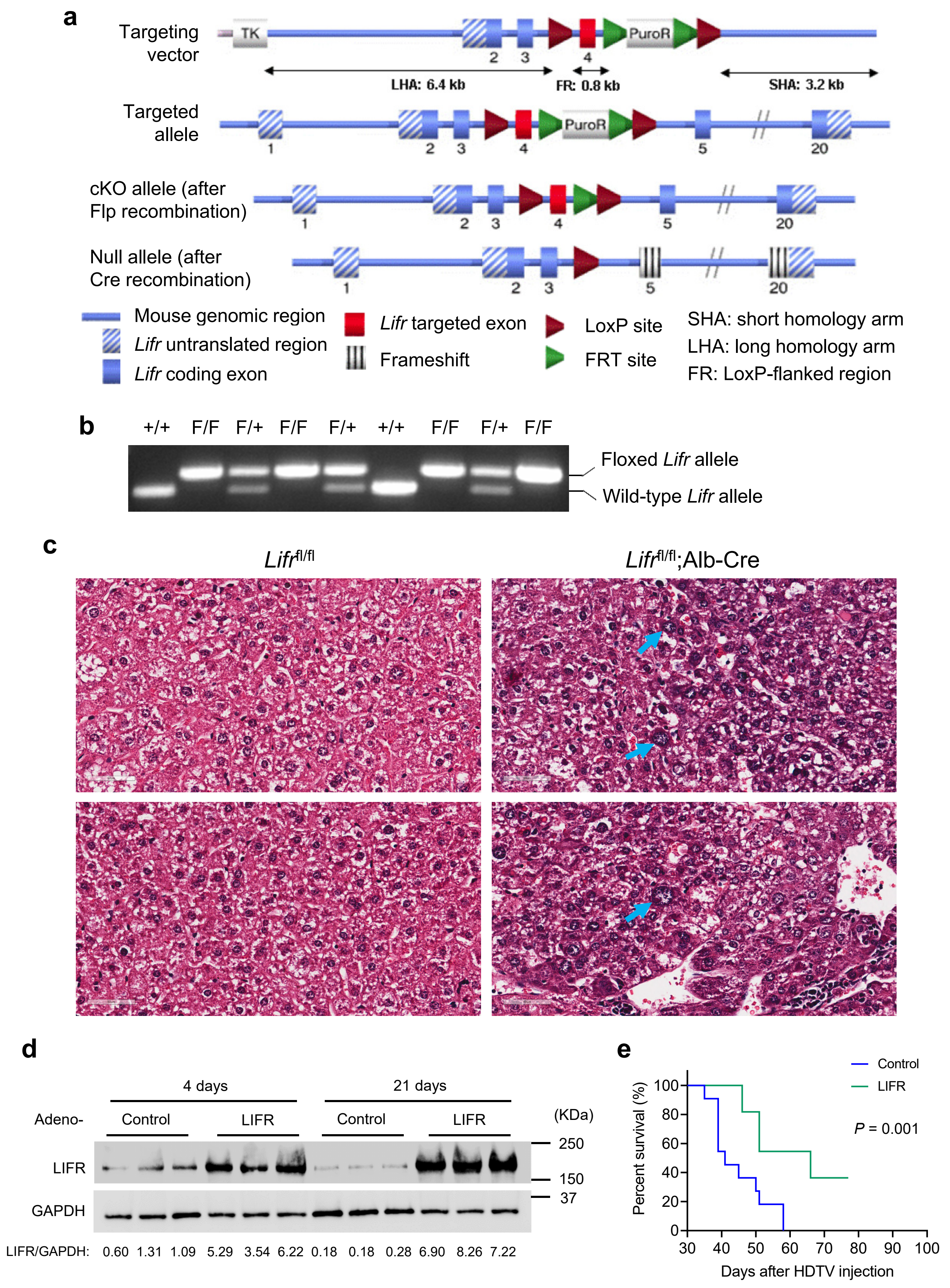

Supplementary Fig. 2. Liver-specific knockout of Lifr in mice and adenoviral delivery of LIFR to the mouse liver.

(**a**) Schematic representation of the *Lifr* gene-targeting strategy. Deletion of exon 4 results in inactivation of the *Lifr* gene by deleting part of the first fibronectin type-III domain and by generating a frameshift.

(**b**) PCR genotyping results of pups obtained from intercrossing between *Lifr*$^{\text{flox/+}}$ heterozygotes. F/+: *Lifr*$^{\text{flox/+}}$; F/F: *Lifr*$^{\text{flox/flox}}$.

(**c**) H&E staining of livers from DEN-treated *Lifr*$^{\text{fl/fl}}$ and *Lifr*$^{\text{fl/fl}}$;Alb-Cre mice at 7 months of age. Blue arrows indicate nuclear atypia. Scale bars, 50 μm.

(**d**) Immunoblotting of LIFR and GAPDH in livers of C57BL/6 mice that received control adenovirus or LIFR-expressing adenovirus administration 3 days and 17 days after hydrodynamic injection of plasmids expressing the Sleeping Beauty transposase, myrAKT, and RasV12. The tissues were collected at 4 days and 21 days after the first adenovirus administration.

(**e**) Kaplan-Meier curves of overall survival of C57BL/6 mice that received control adenovirus or LIFR-expressing adenovirus administration 3 days and 17 days after hydrodynamic injection of plasmids expressing the Sleeping Beauty transposase, myrAKT, and RasV12. Statistical significance was determined by a log-rank test. $n = 11$ mice.

Source data are provided as a Source Data file.

Supplementary Fig. 3

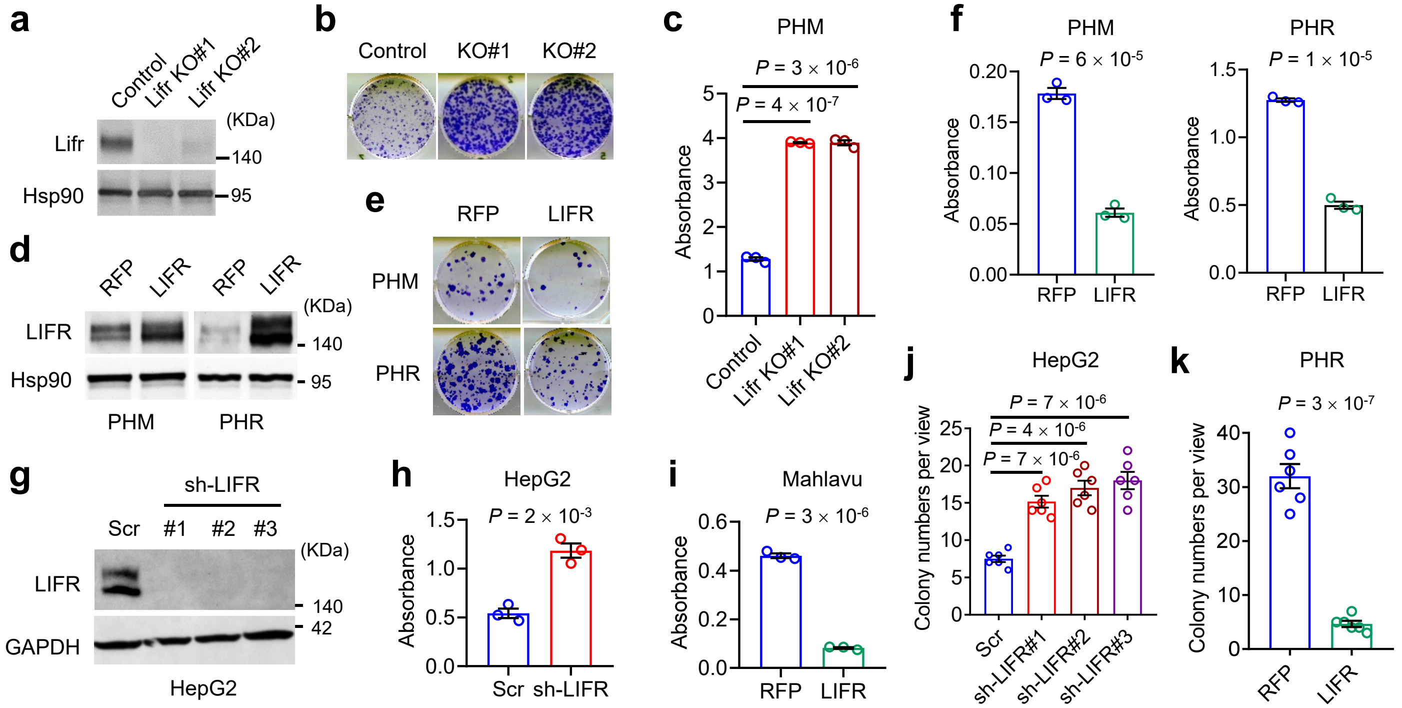

Supplementary Fig. 3. LIFR inhibits the growth of mouse and human liver cells.

(**a**) Immunoblotting of Lifr and Hsp90 in Lifr-knockout PHM cells generated by CRISPR-Cas9.

(**b, c**) Images (**b**; crystal violet staining) and quantification (**c**; gauged by absorbance) of colonies formed by Lifr-knockout PHM cells. *n* = 3 wells.

(**d**) Immunoblotting of Lifr and Hsp90 in PHM and PHR cells transduced with LIFR or an RFP control.

(**e, f**) Images (**e**) and quantification (**f**) of colonies formed by LIFR-overexpressing PHM and PHR cells. *n* = 3 wells.

(**g**) Immunoblotting of LIFR and GAPDH in LIFR-knockdown HepG2 cells.

(**h, i**) Quantification of colonies formed by LIFR-knockdown HepG2 cells (**h**) and LIFR-overexpressing Mahlavu cells (**i**). *n* = 3 wells.

(**j, k**) Quantification of colonies formed by LIFR-knockdown HepG2 (**j**) and LIFR-overexpressing PHR (**k**) cells in soft agar. *n* = 6 wells.

In all *in vitro* assays, cells were seeded at clonogenic densities. Statistical significance in **c**, **f**, and **h**-**k** was determined by a two-tailed unpaired *t*-test. Error bars are s.e.m. Source data are provided as a Source Data file.

Supplementary Fig. 4

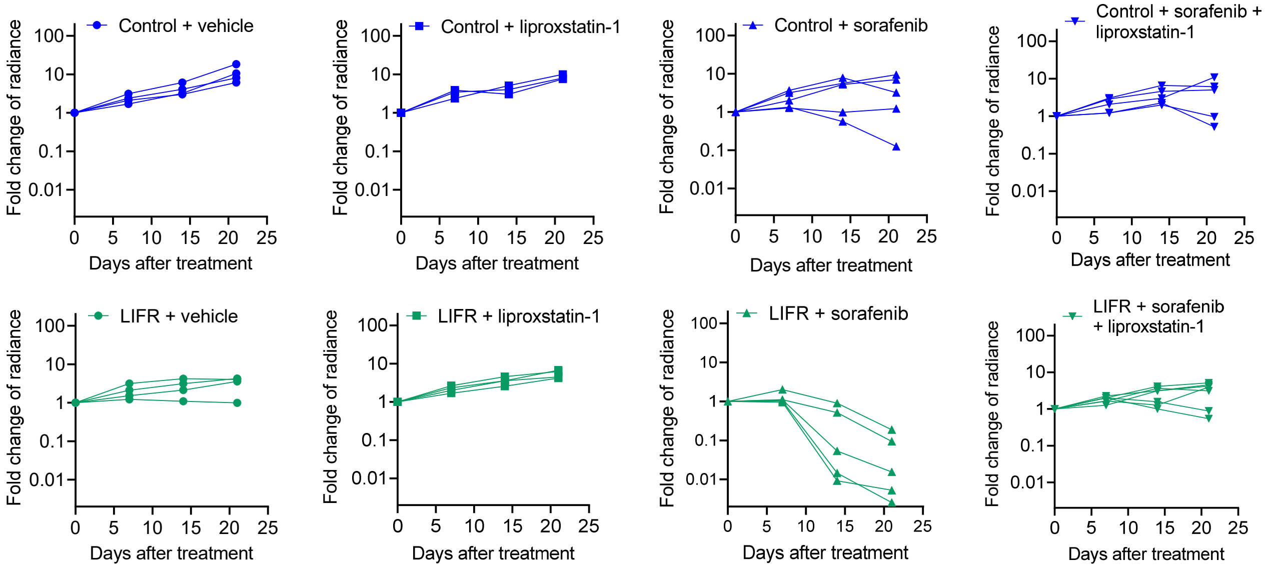

Control + vehicle *versus* LIFR + vehicle: *P* = 0.034

LIFR + sorafenib *versus* LIFR + sorafenib + liporoxstatin-1: *P* = 0.0034

Control + sorafenib *versus* LIFR + sorafenib: *P* = 0.046

Supplementary Fig. 4. LIFR enhances the anti-tumor effect of sorafenib in a ferroptosis-dependent manner.

Quantification of the photon flux of C57BL/6 mice that received control adenovirus or LIFR-expressing adenovirus administration 3 days and 17 days after hydrodynamic injection of plasmids expressing the Sleeping Beauty transposase, myrAKT-IRES-luciferase, and RasV12. One week after plasmid injection, mice received 30 mg kg^{-1} sorafenib and/or 10 mg kg^{-1} liproxstatin-1. The treatments were given 6 days a week. Statistical significance was determined by a two-tailed unpaired *t*-test. Upper panels: *n* = 4, 3, 5, and 5 mice from left to right. Lower panels: *n* = 4, 4, 5, and 7 mice from left to right. Source data are provided as a Source Data file.

Supplementary Fig. 5

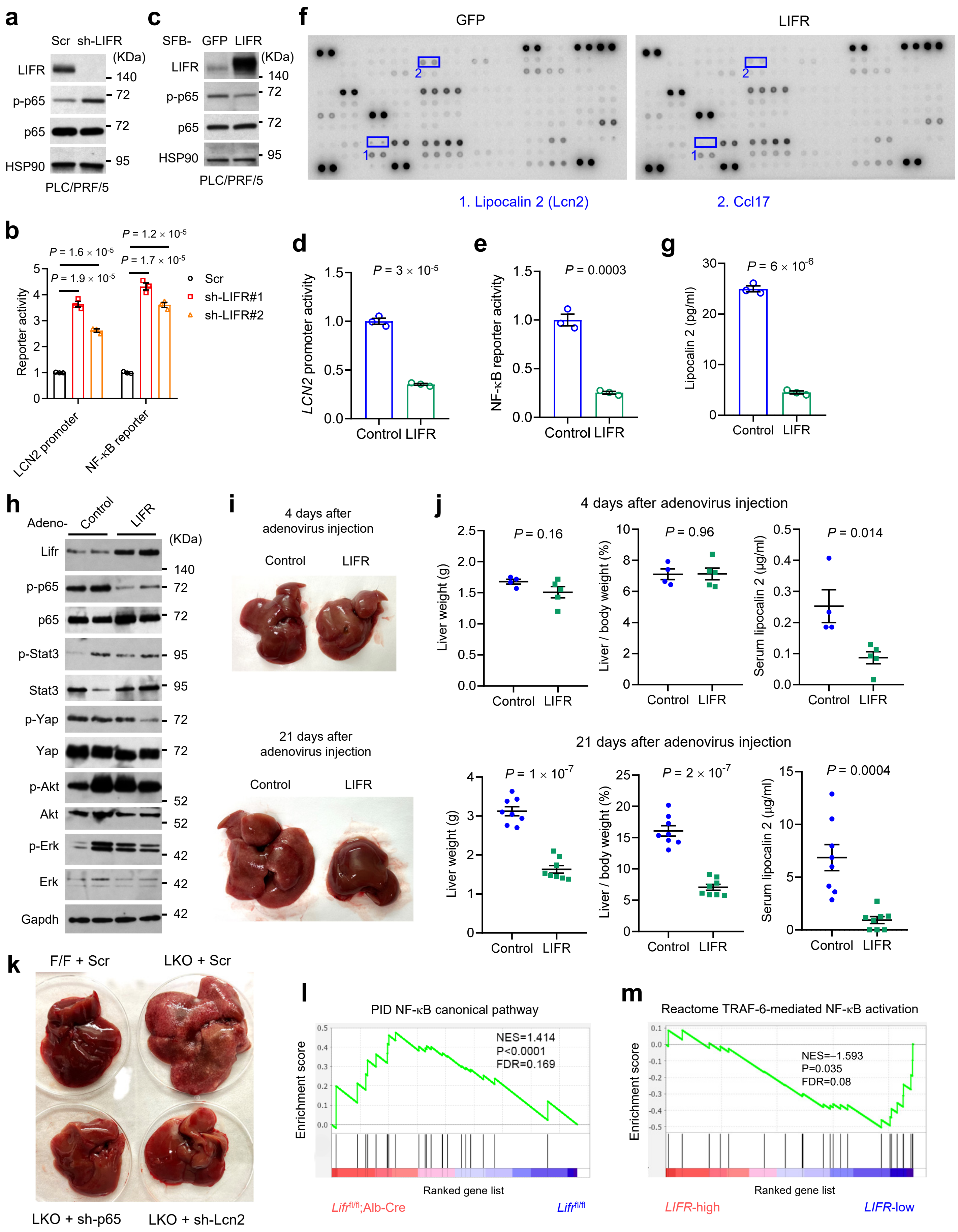

Supplementary Fig. 5. LIFR inhibits NF-κB signaling and downregulates Lcn2.

(**a**) Immunoblotting of LIFR, p-p65, p65, and HSP90 in LIFR-knockdown PLC/PRF/5 cells.

(**b**) Luciferase activity in control or LIFR-knockdown HEK293T cells co-transfected with Renilla luciferase and a firefly luciferase reporter containing the human *LCN2* promoter or the NF-κB-binding site. $n = 3$ wells.

(**c**) Immunoblotting of LIFR, p-p65, p65, and HSP90 in LIFR-overexpressing PLC/PRF/5 cells.

(**d, e**) Luciferase activity in HEK293T cells co-transfected with LIFR, Renilla luciferase, and a firefly luciferase reporter containing the human *LCN2* promoter (**d**) or the NF-κB-binding site (**e**). $n = 3$ wells.

(**f**) Cytokine arrays of the conditioned medium of control and LIFR-overexpressing PHM cells. Boxed: downregulated (blue) cytokines in the LIFR-overexpressing group.

(**g**) ELISA of lipocalin 2 in the conditioned medium of LIFR-overexpressing PHM cells. $n = 3$ wells.

(**h**) Pathway analysis of livers of C57BL/6 mice that received control or LIFR-expressing adenovirus 3 days and 17 days after hydrodynamic injection of plasmids expressing the Sleeping Beauty transposase, myrAKT, and RasV12.

(**i**) Images of livers from C57BL/6 mice that received control or LIFR-overexpressing adenovirus 3 days and 17 days after hydrodynamic injection of plasmids expressing the Sleeping Beauty transposase, myrAKT, and RasV12. Tissues were collected 4 days and 21 days after the first adenovirus administration.

(**j**) Liver weight, liver-to-body weight ratio, and serum lipocalin 2 levels of the mice described in **i**. Upper panels: $n = 4$ mice in the control group and $n = 5$ mice in the LIFR group. Lower panels: $n = 8$ mice.

(**k**) Images of livers from *Lifr*$^{fl/fl}$ (F/F) and *Lifr*$^{fl/fl}$;Alb-Cre (LKO) mice, 57 days after hydrodynamic injection of plasmids expressing the Sleeping Beauty transposase, myrAKT, RasV12, and shRNA (sh-p65, sh-Lcn2, or scrambled).

(**l**) GSEA of RNA-seq data of liver-specific Lifr-knockout mice.

(**m**) GSEA of microarray data (GSE76427) of human HCC. Statistical significance in **l** and **m** was determined using the GSEA software. NES: normalized enrichment score; FDR: false discovery rate.

Statistical significance in **b**, **d**, **e**, **g**, and **j** was determined by a two-tailed unpaired *t*-test. Error bars are s.e.m. Source data are provided as a Source Data file.

Supplementary Fig. 6

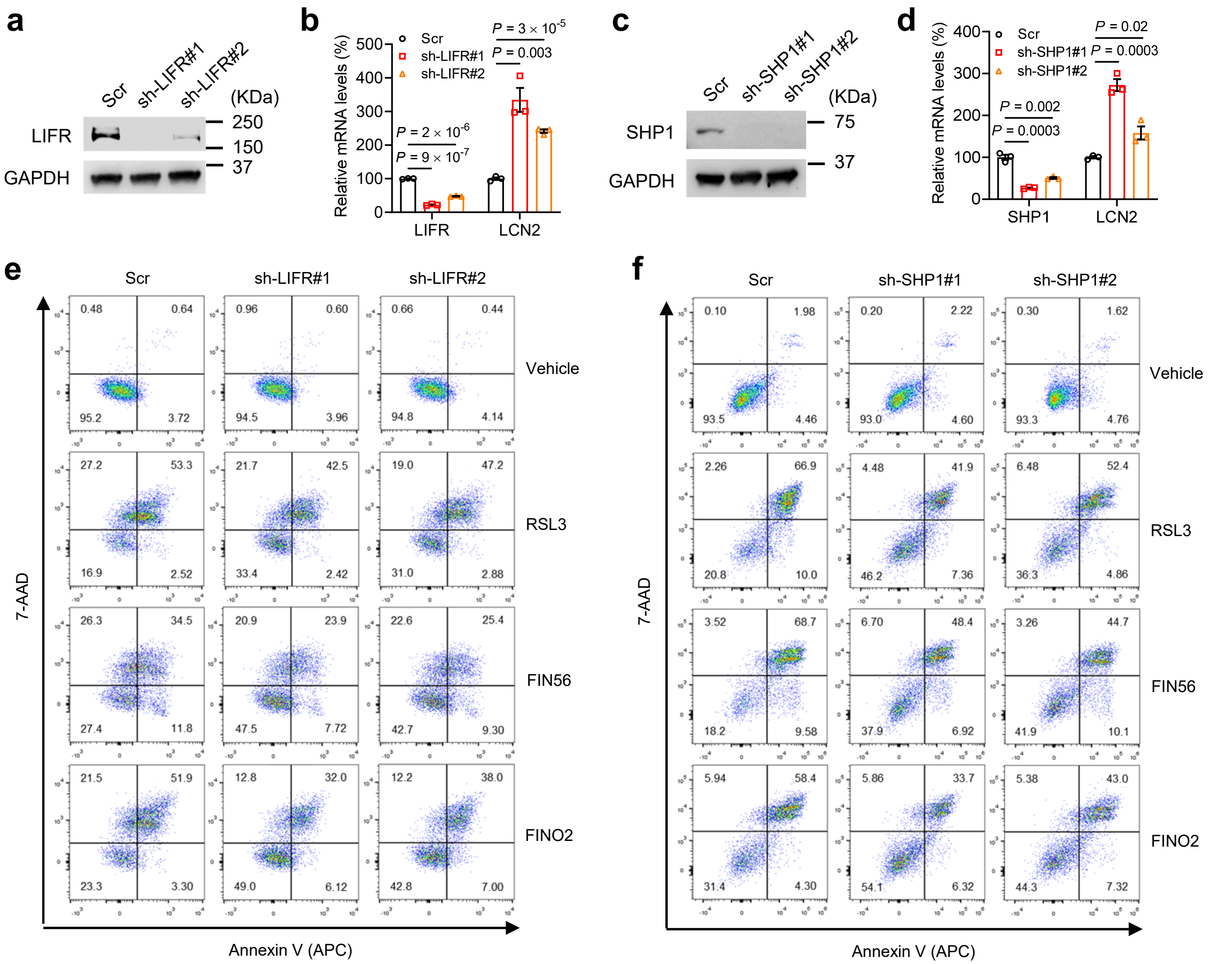

Supplementary Fig. 6. Knockdown of LIFR or SHP1 leads to upregulation of *LCN2* and protects against cell death induced by RSL3, FIN56, and FINO2.

(**a**) Immunoblotting of LIFR and GAPDH in HT1080 cells transduced with scrambled control shRNA (Scr) or LIFR shRNA.

(**b**) qPCR analysis of *LIFR* and *LCN2* in HT1080 cells transduced with control shRNA or LIFR shRNA.

(**c**) Immunoblotting of SHP1 and GAPDH in HT1080 cells transduced with control shRNA or SHP1 shRNA.

(**d**) qPCR analysis of *SHP1* and *LCN2* in HT1080 cells transduced with control shRNA or SHP1 shRNA.

(**e**) HT1080 cells transduced with scrambled control shRNA (Scr) or LIFR shRNA were treated with 0.5 µM RSL3 for 12 h, 50 µM FIN56 for 6 h, or 10 µM FINO2 for 24 h. Plots show staining of 7-aminoactinomycin (7-AAD) and annexin V.

(**f**) HT1080 cells transduced with control shRNA or SHP1 shRNA were treated with 0.5 µM RSL3 for 12 h, 50 µM FIN56 for 6 h, or 10 µM FINO2 for 24 h. Plots show staining of 7-AAD and annexin V.

Statistical significance in **b** and **d** was determined by a two-tailed unpaired *t*-test. Error bars are s.e.m. $n = 3$ samples. Source Data are provided as a Source Data file.

Supplementary Fig. 7

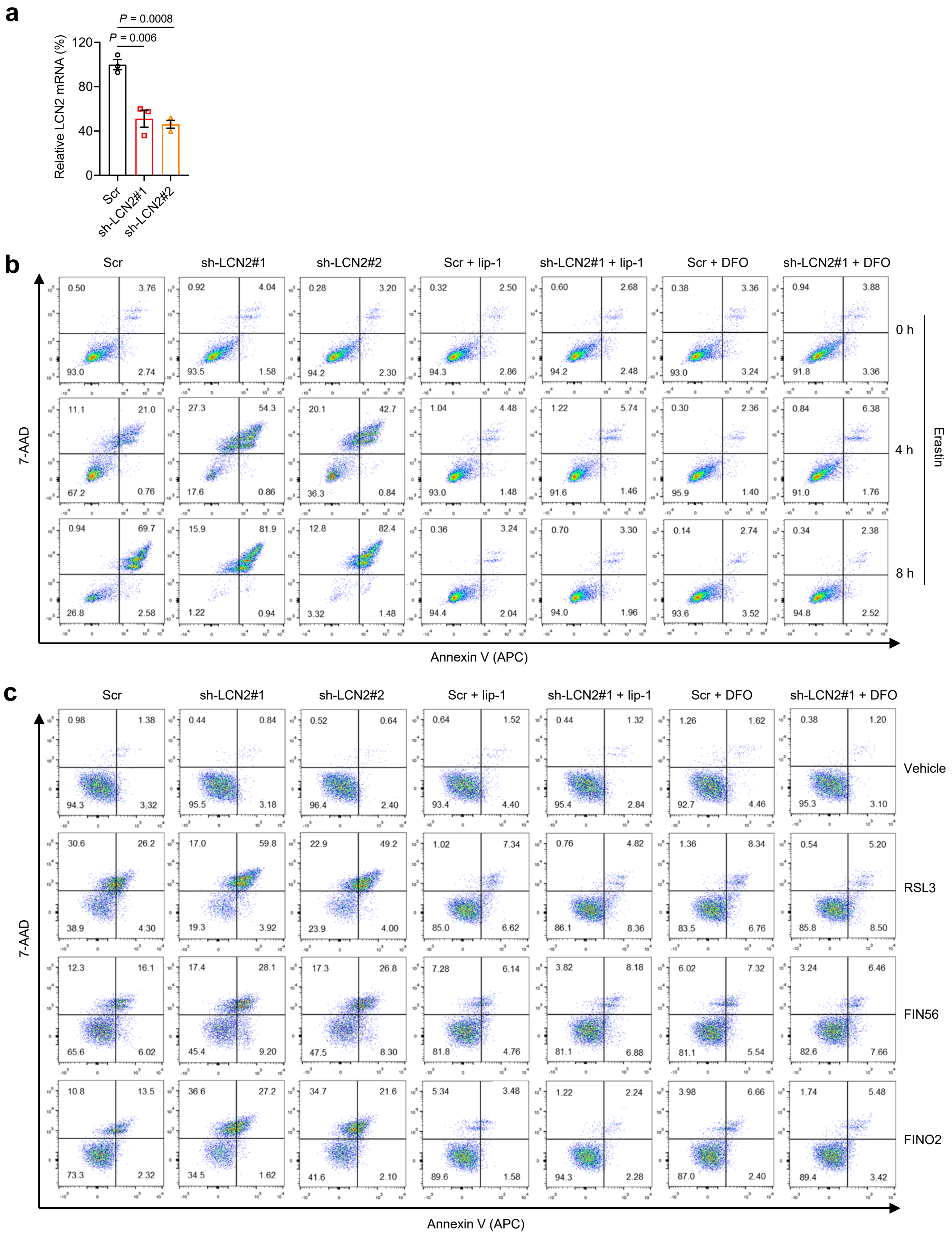

Supplementary Fig. 7. Knockdown of LCN2 sensitizes cells to death induced by erastin, RSL3, FIN56, and FINO2 in a ferroptosis-dependent manner.

(**a**) qPCR analysis of *LCN2* in HT1080 cells transduced with scrambled control shRNA (Scr) or LCN2 shRNA. Statistical significance was determined by a two-tailed unpaired *t*-test. Error bars are s.e.m. $n = 3$ samples.

(**b**) HT1080 cells transduced with control shRNA or LCN2 shRNA were treated with 10 μM erastin for 0, 4, or 8 h, alone or in combination with liproxstatin-1 (lip-1, 10 μM) or DFO (100 μM). Plots show staining of 7-aminoactinomycin (7-AAD) and annexin V.

(**c**) HT1080 cells transduced with scrambled control shRNA or LCN2 shRNA were treated with 0.5 μM RSL3 for 10 h, 50 μM FIN56 for 3 h, or 10 μM FINO2 for 12 h, alone or in combination with liproxstatin-1 (lip-1, 10 μM) or DFO (100 μM). Plots show staining of 7-aminoactinomycin (7-AAD) and annexin V.

Source data are provided as a Source Data file.

Supplementary Fig. 8

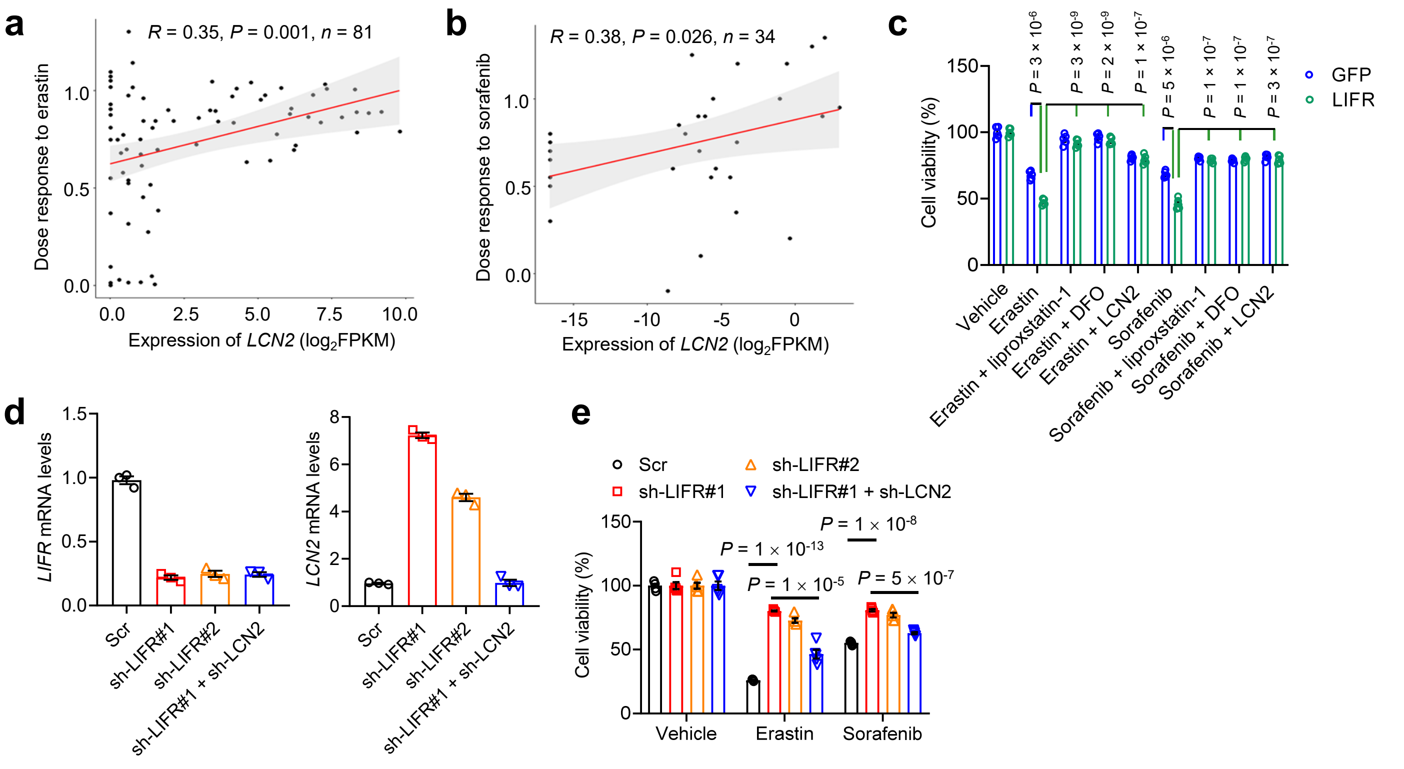

Supplementary Fig. 8. LCN2 mediates ferroptosis resistance.

(**a**) Correlation between *LCN2* expression and dose response to erastin, based on the liver cancer cell lines (n = 81) from the LIMORE dataset.

(**b**) Correlation between *LCN2* expression and dose response to sorafenib, based on the human liver cancer cell lines (n = 34) from the LCCL dataset. Linear relationships in **a** and **b** were determined by the Pearson correlation analysis.

(**c**) Control and LIFR-overexpressing PLC/PRF/5 cells were treated with DMSO (vehicle), erastin (10 μM), or sorafenib (20 μM), alone or in combination with liproxstatin-1 (5 μM), DFO (50 μM) or purified LCN2 (100 μg/ml), and cell viability was measured by a CCK8 assay. n = 5 wells.

(**d**) qPCR of *LIFR* (left panel) and *LCN2* (right panel) in Mahlavu cells transduced with LIFR shRNA alone or in combination with LCN2 shRNA. n = 3 samples.

(**e**) Mahlavu cells were transduced with LIFR shRNA alone or in combination with LCN2 shRNA. The cells were treated with 10 μM erastin or 20 μM sorafenib, and cell viability was determined by a CCK8 assay. n = 5 wells.

Statistical significance in **c** and **e** was determined by a two-tailed unpaired t-test. Error bars are s.e.m. Source data are provided as a Source Data file.

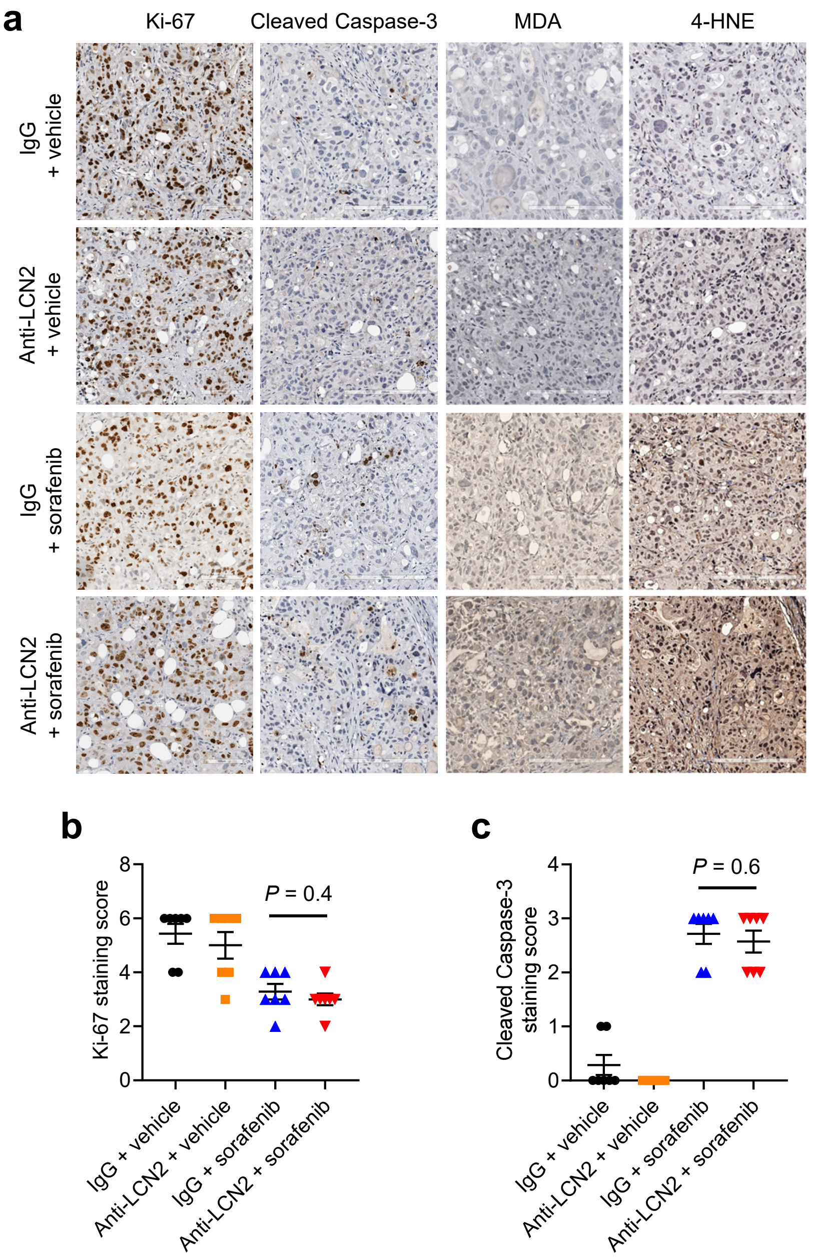

Supplementary Fig. 9. Combination treatment with sorafenib and an LCN2-neutralizing antibody increases lipid peroxidation levels in PDX tumors.

(**a**) Immunohistochemical staining of Ki-67 (in viable tumor areas), cleaved caspase-3, MDA, and 4-HNE in tumor tissues from NSG mice bearing PDX line #5. Mice were treated with anti-LCN2 and sorafenib, alone or in combination. Scale bars, 200 µm.

(**b, c**) Quantification of immunohistochemical staining of Ki-67 (in viable tumor areas; **b**) and cleaved caspase-3 (**c**) in tumor tissues of the mice described in **a**. n = 7 mice. Statistical significance was determined by a one-way ANOVA (to compare the means among three or more groups) and a two-tailed unpaired t-test (to compare the means between two groups). Error bars are s.e.m.

Source data are provided as a Source Data file.

Supplementary Fig. 10

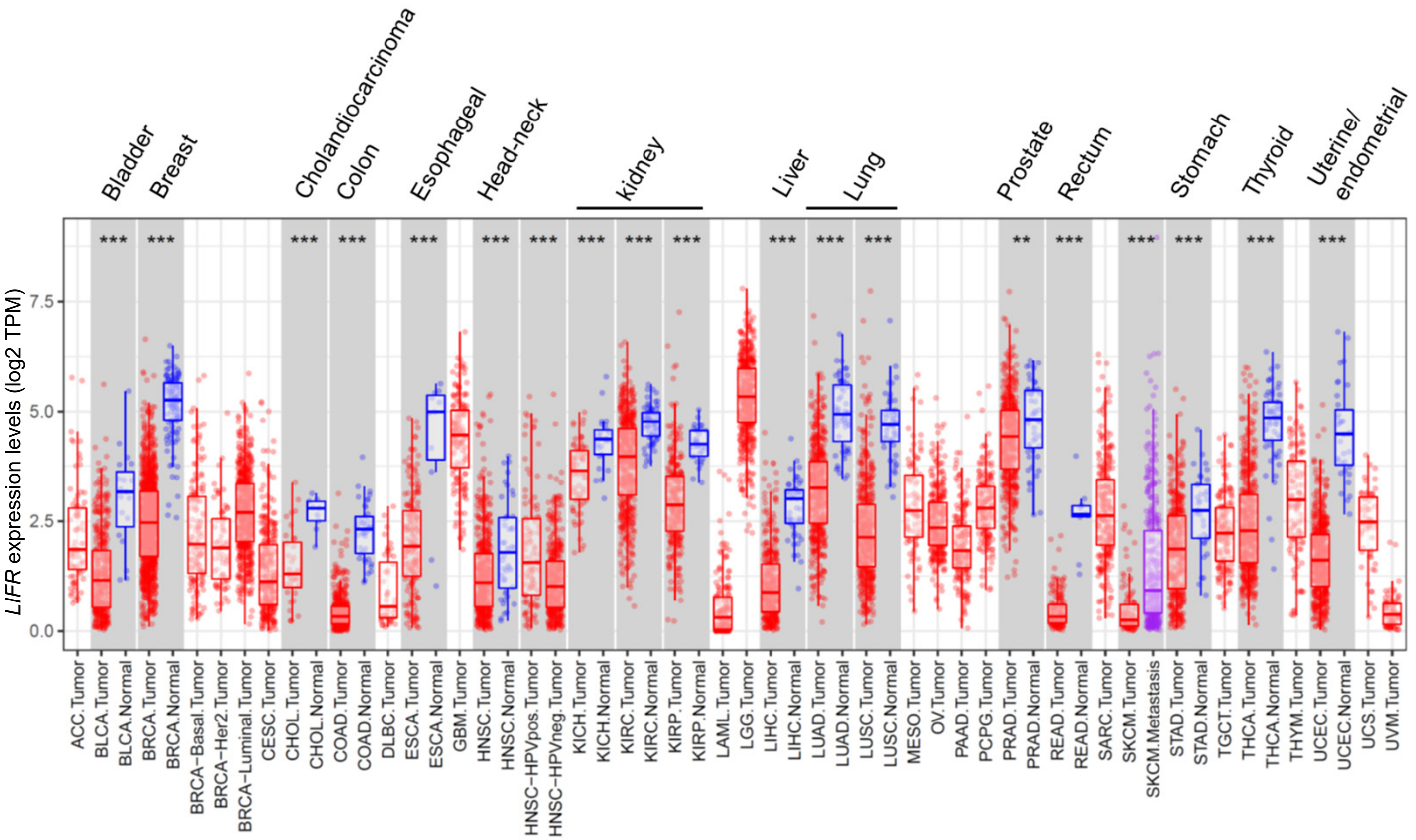

Supplementary Fig. 10. *LIFR* is downregulated in many types of cancer.

LIFR expression levels in different tumor types from the TCGA database were determined by TIMER (https://cistrome.shinyapps.io/timer/). *: *P* < 0.05, **: *P* < 0.01, ***: *P* < 0.001. TPM: transcript per million. ACC: adrenocortical carcinoma. BLCA: bladder urothelial carcinoma. BRCA: breast invasive carcinoma. CESC: cervical squamous cell carcinoma and endocervical adenocarcinoma. CHOL: cholangiocarcinoma. COAD: colon adenocarcinoma. DLBC: lymphoid neoplasm diffuse large B-cell lymphoma. ESCA: esophageal carcinoma. GBM: glioblastoma multiforme. HNSC: head and neck squamous cell carcinoma. KICH: kidney chromophobe. KIRC: kidney renal clear cell carcinoma. KIRP: kidney renal papillary cell carcinoma. LAML: acute myeloid leukemia. LGG: brain lower-grade glioma. LIHC: liver hepatocellular carcinoma. LUAD: lung adenocarcinoma. LUSC: lung squamous cell carcinoma. MESO: mesothelioma. OV: ovarian serous cystadenocarcinoma. PAAD: pancreatic adenocarcinoma. PCPG: pheochromocytoma and paraganglioma. PRAD: prostate adenocarcinoma. READ: rectum adenocarcinoma. SARC: sarcoma. SKCM: skin cutaneous melanoma. STAD: stomach adenocarcinoma. TGCT: testicular germ cell tumors. THCA: thyroid carcinoma. THYM: thymoma. UCEC: uterine corpus endometrial carcinoma. UCS: uterine carcinosarcoma. UVM: uveal melanoma.

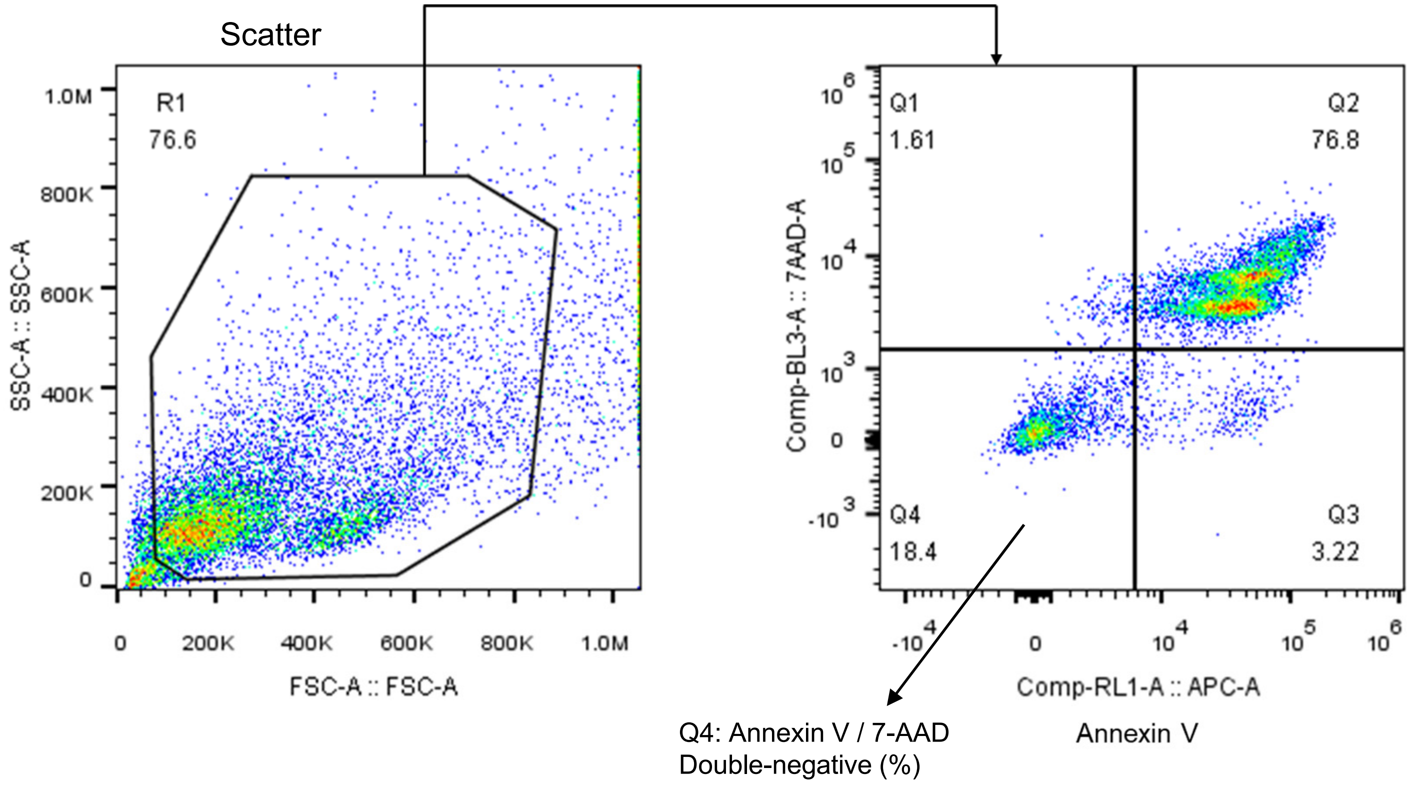

Supplementary Fig. 11. An example of the gating strategy for annexin V and 7-AAD analysis by flow cytometry. Initial cell population gating (SSC-Area vs FSC-Area) was adopted to ensure that only single cells were used for analysis.

Supplementary Table 1. Primer and shRNA sequence information

Use	Gene	Direction	Sequence (5' → 3')
Human RNA qPCR	*ACTB* (for normalization)	Forward	GATCATTGCTCCTCCTGAGC
		Reverse	ACTCCTGCTTGCTGATCCAC
	LIFR		Sigma-Aldrich Predesigned SYBR Green primer for RT-qPCR, RefSeq ID: NM_001127671
	LCN2		Bio-Rad PrimePCR[TM] PreAmp for SYBR Green Assay, human *LCN2*
	RELA (encoding p65)	Forward	TCAATGGCTACACAGGACCA
		Reverse	ATCTTGAGCTCGGCAGTGTT
	PTPN6 (encoding SHP1)	Forward	GGAGAAGTTTGCGACTCTGAC
		Reverse	GCGGGTACTTGAGGTGGATG
Mouse RNA qPCR	*Gapdh* (for normalization)	Forward	TCACCACCATGGAGAAGGC
		Reverse	GCTAAGCAGTTGGTGGTGCA
	Lifr		Sigma-Aldrich Predesigned SYBR Green primer for RT-qPCR, RefSeq ID: NM_001113386
	Lcn2		Bio-Rad PrimePCR[TM] PreAmp for SYBR Green Assay, mouse *Lcn2*
	RelA	Forward	TGACCCCTGTCCTCTCACATCCG
		Reverse	CAGCTCCCAGAGTTCCGGTT
Genotyping	Cre-ERT2 transgene	Forward	CGGTCGATGCAACGAGTGAT
		Reverse	CCACCGTCAGTACGTGAGAT
	Lifr allele (*)	Forward	CAGTGGTTATCTGTGAGACTCAGG
		Reverse	GGAGTCAATGGCGATATGAGG
	Albumin-Cre transgene	Wild-type Forward	TGCAAACATCACATGCACAC
		Mutant Forward	GAAGCAGAAGCTTAGGAAGATGG

		Common Reverse	TTGGCCCCTTACCATAACTG
Human shRNA	*LIFR*	#1	GCTCCCATTGTTGCACCAAAT
		#2	CCACCCATCATTGAGGAAGAA
		#3	CCTCAGATATGCCCTTGGAAT
	LCN2	#1	CCAGCATGCTATGGTGTTCTT
		#2	GTACTTCAAGATCACCCTCTA
	PTPN6 (encoding SHP1)	#1	GGAGCATGACACAACCGAATA
		#2	CCTCTCCCTGACCCTGTATAT
	RELA (encoding p65)	#1	CACCATCAACTATGATGAGTT
Mouse shRNA	*Lcn2*	#1	TTTCTGGACCGCATTGCCT
		#2	TTGTAGCTATTGTTCTCTT
	RelA (encoding p65)	#1	AGGCCATATAGCCTTACTATC
	Lcn2	Sleeping beauty vector	TGCCACTCCATCTTTCCTGTT
	RelA (encoding p65)	Sleeping beauty vector	GGAGTACCCTGAAGCTATAAC
	Scrambled control	Sleeping beauty vector	CCTAAGGTTAAGTCGCCCTCG

(*) The PCR product is 356 bp for the wild-type *Lifr* allele and is 418 bp for the LoxP-flanked *Lifr* allele (*Lifr*^{flox}). See **Supplementary Fig. 2b**.
