# Supplementary material for: A targetable LIFR−NF-κB−LCN2 axis controls liver tumorigenesis and vulnerability to ferroptosis
Source: Nat Commun. 2021 Dec 17;12:7333. doi: 10.1038/s41467-021-27452-9 (PMC8683481; doi:10.1038/s41467-021-27452-9)
Supplement: Supplementary file 2 — Description of Additional Supplementary Files [file 41467_2021_27452_MOESM2_ESM.pdf]

Description of Additional Supplementary Files

File Name: Supplementary Data 1
Description: Genes that are downregulated or upregulated after hepatocyte-specific knockout of Lifr
