# Supplementary material for: A targetable LIFR−NF-κB−LCN2 axis controls liver tumorigenesis and vulnerability to ferroptosis
Source: Nat Commun. 2021 Dec 17;12:7333. doi: 10.1038/s41467-021-27452-9 (PMC8683481; doi:10.1038/s41467-021-27452-9)
Supplement: Supplementary file 4 — Reporting Summary [file 41467_2021_27452_MOESM4_ESM.pdf]

nature portfolio

Corresponding author(s): Li Ma

Last updated by author(s): Nov 7, 2021

Reporting Summary

Nature Portfolio wishes to improve the reproducibility of the work that we publish. This form provides structure for consistency and transparency in reporting. For further information on Nature Portfolio policies, see our Editorial Policies and the Editorial Policy Checklist.

Statistics

For all statistical analyses, confirm that the following items are present in the figure legend, table legend, main text, or Methods section.

n/a	Confirmed	
☐	☒	The exact sample size (*n*) for each experimental group/condition, given as a discrete number and unit of measurement
☐	☒	A statement on whether measurements were taken from distinct samples or whether the same sample was measured repeatedly
☐	☒	The statistical test(s) used AND whether they are one- or two-sided<br>*Only common tests should be described solely by name; describe more complex techniques in the Methods section.*
☒	☐	A description of all covariates tested
☐	☒	A description of any assumptions or corrections, such as tests of normality and adjustment for multiple comparisons
☐	☒	A full description of the statistical parameters including central tendency (e.g. means) or other basic estimates (e.g. regression coefficient) AND variation (e.g. standard deviation) or associated estimates of uncertainty (e.g. confidence intervals)
☐	☒	For null hypothesis testing, the test statistic (e.g. *F*, *t*, *r*) with confidence intervals, effect sizes, degrees of freedom and *P* value noted<br>*Give P values as exact values whenever suitable.*
☒	☐	For Bayesian analysis, information on the choice of priors and Markov chain Monte Carlo settings
☒	☐	For hierarchical and complex designs, identification of the appropriate level for tests and full reporting of outcomes
☐	☒	Estimates of effect sizes (e.g. Cohen's *d*, Pearson's *r*), indicating how they were calculated

Our web collection on statistics for biologists contains articles on many of the points above.

Software and code

Policy information about availability of computer code

Data collection	Luciferase imaging of live animals and data collection were performed by using the IVIS-200 Bioluminescence Imaging System (Perkin Elmer). Cells were processed on an Invitrogen Attune NxT Acoustic Focusing Cytometer.
Data analysis	Living Image® software (Perkin Elmer, for the Xenogen IVIS-200 Imaging System) was used for bioluminescent image analysis and data quantification. Cells were processed on an Invitrogen Attune NxT Acoustic Focusing Cytometer and analyzed by FlowJo software (version 10.4).

For manuscripts utilizing custom algorithms or software that are central to the research but not yet described in published literature, software must be made available to editors and reviewers. We strongly encourage code deposition in a community repository (e.g. GitHub). See the Nature Portfolio guidelines for submitting code & software for further information.

Data

Policy information about availability of data

All manuscripts must include a data availability statement. This statement should provide the following information, where applicable:

- Accession codes, unique identifiers, or web links for publicly available datasets
- A description of any restrictions on data availability
- For clinical datasets or third party data, please ensure that the statement adheres to our policy

The source data that support the findings of this study are available. The RNA-seq raw data have been deposited at the Gene Expression Omnibus (GEO) under the accession number GSE177042 (https://www.ncbi.nlm.nih.gov/geo/query/acc.cgi?acc=GSE177042). Source data are provided with this paper.

Field-specific reporting

Please select the one below that is the best fit for your research. If you are not sure, read the appropriate sections before making your selection.

☒ Life sciences ☐ Behavioural & social sciences ☐ Ecological, evolutionary & environmental sciences

For a reference copy of the document with all sections, see nature.com/documents/nr-reporting-summary-flat.pdf

Life sciences study design

All studies must disclose on these points even when the disclosure is negative.

Sample size	Based on the literature and our previous studies (PMID: 34188037; PMID: 29891922; PMID: 30349115), we chose the sample size routinely used for animal experiments, reporter assays, quantitative PCR, soft agar assays, cell death and viability assays, and ELISA.
Data exclusions	No data were excluded.
Replication	Except for the animal studies (one time), RNA-seq (one time), and cytokine array (one time), each experiment was repeated at least three times with similar results.
Randomization	Mice were randomly assigned to different treatment groups.
Blinding	For cell-based experiments, blinding was not performed, because the investigator had to know the groups to load the samples or perform the assay. Blinding was not performed in mouse experiments, because the investigator needed to know the treatment groups in order to perform the study.

Reporting for specific materials, systems and methods

We require information from authors about some types of materials, experimental systems and methods used in many studies. Here, indicate whether each material, system or method listed is relevant to your study. If you are not sure if a list item applies to your research, read the appropriate section before selecting a response.

Materials & experimental systems

n/a	Involved in the study	
☐	☒	Antibodies
☐	☒	Eukaryotic cell lines
☒	☐	Palaeontology and archaeology
☐	☒	Animals and other organisms
☐	☒	Human research participants
☒	☐	Clinical data
☒	☐	Dual use research of concern

Methods

n/a	Involved in the study	
☒	☐	ChIP-seq
☐	☒	Flow cytometry
☒	☐	MRI-based neuroimaging

Antibodies

Antibodies used

-Antibodies used for immunoblotting:
Anti-LIFR, Proteintech Cat# 22779-1-AP, RRID: AB_2879165
Anti-GAPDH, ThermoFisher Scientific Cat# MA5-15738, RRID: AB_10977387
Anti-HSP90, BD Biosciences Cat# 610419, RRID: AB_397799
Anti-FLAG, Sigma-Aldrich Cat# F7425, RRID: AB_439687
Anti-p-p65, Cell Signaling Technology Cat# 3033, RRID: AB_331284
Anti-p65, Cell Signaling Technology Cat# 6956, RRID: AB_10828935
Anti-p-STAT3, Cell Signaling Technology Cat# 9145, RRID: AB_2491009
Anti-STAT3, Cell Signaling Technology Cat# 9139, RRID: AB_331757
Anti-p-YAP, Cell Signaling Technology Cat# 4911, RRID: AB_2218913
Anti-YAP, Cell Signaling Technology Cat# 14074, RRID: AB_2650491
Anti-p-AKT, Santa Cruz Biotechnology Cat# sc-7985, RRID: AB_667741
Anti-AKT, Cell Signaling Technology Cat# 2920, RRID: AB_1147620
Anti-p-ERK, Cell Signaling Technology Cat# 9101, RRID: AB_331646
Anti-ERK, Cell Signaling Technology Cat# 4696, RRID: AB_390780
Anti-TRAF6, Cell Signaling Technology Cat# 8028, RRID: AB_10858223
Anti-SHP1, Cell Signaling Technology Cat# 3759, RRID: AB_2173694
Anti-SHP2, Cell Signaling Technology Cat# 3397, RRID: AB_2174959
Anti-Xpress, ThermoFisher Scientific Cat# R910-25, RRID: AB_2556552
Anti-LCN2, R&D Systems Cat# AF1757, RRID: AB_354974
Anti-GPX4, R&D Systems Cat# MAB5457, RRID: AB_2232542

Anti-FSP1, Proteintech Cat#20886-1-AP, RRID: AB_2878756
Anti-SLC7A11, Cell Signaling Technology Cat# 98051, RRID: AB_2800296
Anti-p-IKKa/b, Cell signaling technology Cat# 2697s, RRID: AB_2079382
Anti-IKKb, Cell signaling technology Cat#8943s, RRID: AB_11024092
Anti-IkBa, Cell signaling technology Cat#4812s, RRID: AB_10694416

-Antibodies used for IHC:
Anti-4-HNE, Abcam Cat# ab46545, RRID: AB_722490
Anti-MDA, AdipoGen Cat# JAI-MMD-030N
Anti-Ki-67, Cell Signaling Technology Cat# 9027, RRID: AB_2636984
Anti-cleaved caspase 3, Cell Signaling Technology Cat# 9661, RRID: AB_2341188
Anti-LCN2, R&D Systems Cat# AF1857, RRID: AB_355022
goat IgG HRP-conjugated antibody, R&D systems, HAF017, RRID:AB_562588

-Antibodies used for treating mice with PDX tumors:
IgG, R&D Systems, Cat# MAB006-MTO, clone# 54447
Anti-LCN2, R&D Systems, Cat# MAB1757-MTO, clone# 220310

Validation

Pre-validated antibodies were purchased from reputable sources. All proteins are well studied and all antibodies are widely used in the literature. We validated the antibodies for endogenous and transfected proteins in knockdown, knockout, and overexpression settings.

-Antibodies used for immunoblotting:
Anti-LIFR, Proteintech Cat# 22779-1-AP, RRID: AB_2879165 https://www.ptglab.com/products/LIFR-Antibody-22779-1-AP.htm
Anti-GAPDH, ThermoFisher Scientific Cat# MA5-15738, RRID: AB_10977387 https://www.thermofisher.com/antibody/product/GAPDH-Loading-Control-Antibody-clone-GA1R-Monoclonal/MA5-15738
Anti-HSP90, BD Biosciences Cat# 610419, RRID: AB_397799 https://www.bdbiosciences.com/en-us/products/reagents/microscopy-imaging-reagents/immunofluorescence-reagents/purified-mouse-anti-hsp90.610419
Anti-FLAG, Sigma-Aldrich Cat# F7425, RRID: AB_439687 https://www.cellsignal.com/products/primary-antibodies/cbp-d9b6-rabbit-mab/7425
Anti-p-p65, Cell Signaling Technology Cat# 3033, RRID: AB_331284 https://www.cellsignal.com/products/primary-antibodies/phospho-nf-kb-p65-ser536-93h1-rabbit-mab/3033
Anti-p65, Cell Signaling Technology Cat# 6956, RRID: AB_10828935 https://www.cellsignal.com/products/primary-antibodies/nf-kb-p65-l8f6-mouse-mab/6956
Anti-p-STAT3, Cell Signaling Technology Cat# 9145, RRID: AB_2491009 https://www.cellsignal.com/products/primary-antibodies/phospho-stat3-tyr705-d3a7-xp-rabbit-mab/9145
Anti-STAT3, Cell Signaling Technology Cat# 9139, RRID: AB_331757 https://www.cellsignal.com/products/primary-antibodies/stat3-124h6-mouse-mab/9139
Anti-p-YAP, Cell Signaling Technology Cat# 4911, RRID: AB_2218913 https://www.cellsignal.com/products/primary-antibodies/phospho-yap-ser127-antibody/4911
Anti-YAP, Cell Signaling Technology Cat# 14074, RRID: AB_2650491 https://www.cellsignal.com/products/primary-antibodies/yap-d8h1x-xp-rabbit-mab/14074
Anti-p-AKT, Santa Cruz Biotechnology Cat# sc-7985, RRID: AB_667741 https://www.scbt.com/p/p-akt1-2-3-antibody-ser-473-human-alt2
Anti-AKT, Cell Signaling Technology Cat# 2920, RRID: AB_1147620 https://www.cellsignal.com/products/primary-antibodies/akt-pan-40d4-mouse-mab/2920
Anti-p-ERK, Cell Signaling Technology Cat# 9101, RRID: AB_331646 https://www.cellsignal.com/products/primary-antibodies/phospho-p44-42-mapk-erk1-2-thr202-tyr204-antibody/9101
Anti-ERK, Cell Signaling Technology Cat# 4696, RRID: AB_390780 https://www.cellsignal.com/products/primary-antibodies/p44-42-mapk-erk1-2-l34f12-mouse-mab/4696
Anti-TRAF6, Cell Signaling Technology Cat# 8028, RRID: AB_10858223 https://www.cellsignal.com/products/primary-antibodies/traf6-d21g3-rabbit-mab/8028
Anti-SHP1, Cell Signaling Technology Cat# 3759, RRID: AB_2173694 https://www.cellsignal.com/products/primary-antibodies/shp-1-c14h6-rabbit-mab/3759
Anti-SHP2, Cell Signaling Technology Cat# 3397, RRID: AB_2174959 https://www.cellsignal.com/products/primary-antibodies/shp-2-d50f2-rabbit-mab/3397
Anti-Xpress, ThermoFisher Scientific Cat# R910-25, RRID: AB_2556552 https://www.thermofisher.com/antibody/product/Xpress-Antibody-Monoclonal/R910-25
Anti-LCN2, R&D Systems Cat# AF1757, RRID: AB_354974 https://www.rndsystems.com/products/human-mouse-rat-lipocalin-2-ngal-antibody_af1757
Anti-GPX4, R&D Systems Cat# MAB5457, RRID: AB_2232542 https://www.rndsystems.com/products/human-mouse-rat-glutathione-peroxidase-4-gpx4-antibody-565320_mab5457
Anti-FSP1, Proteintech Cat#20886-1-AP, RRID: AB_2878756 https://www.ptglab.com/products/AIFM2-Antibody-20886-1-AP.htm
Anti-SLC7A11, Cell Signaling Technology Cat# 98051, RRID: AB_2800296 https://www.cellsignal.com/products/primary-antibodies/xct-slc7a11-antibody/98051
Anti-p-IKKa/b, Cell signaling technology Cat# 2697s, RRID: AB_2079382 https://www.cellsignal.com/products/primary-antibodies/phospho-ikka-b-ser176-180-16a6-rabbit-mab/2697
Anti-IKKb, Cell signaling technology Cat#8943s, RRID: AB_11024092 https://www.cellsignal.com/products/primary-antibodies/ikkb-d30c6-rabbit-mab/8943
Anti-IkBa, Cell signaling technology Cat#4812s, RRID: AB_10694416 https://www.cellsignal.com/products/primary-antibodies/ikba-44d4-rabbit-mab/4812

-Antibodies used for IHC:
Anti-4-HNE, Abcam Cat# ab46545, RRID: AB_722490 https://www.abcam.com/4-hydroxynonenal-antibody-ab46545.html
Anti-MDA, AdipoGen Cat# JAI-MMD-030N https://adipogen.com/jai-mmd-030n-anti-malondialdehyde-mda-mab-1f83.html/
Anti-Ki-67, Cell Signaling Technology Cat# 9027, RRID: AB_2636984 https://www.cellsignal.com/products/primary-antibodies/ki-67-

d2h10-rabbit-mab-ihc-specific/9027
Anti-cleaved caspase 3, Cell Signaling Technology Cat# 9661, RRID: AB_2341188 https://www.cellsignal.com/products/primary-antibodies/cleaved-caspase-3-asp175-antibody/9661
Anti-LCN2, R&D Systems Cat# AF1857, RRID: AB_355022 https://www.rndsystems.com/products/mouse-lipocalin-2-ngal-antibody_af1857
goat IgG HRP-conjugated antibody, R&D systems, HAF017, RRID:AB_562588 https://www.rndsystems.com/products/goat-igg-hrp-conjugated-antibody_haf017

-Antibodies used for treating mice with PDX tumors:
IgG, R&D Systems, Cat# MAB006-MTO, clone# 54447 https://www.rndsystems.com/products/rat-igg2a-isotype-control_mab006
Anti-LCN2, R&D Systems, Cat# MAB1757-MTO, clone# 220310 https://www.rndsystems.com/products/human-lipocalin-2-ngal-antibody-220310_mab1757

Eukaryotic cell lines

Policy information about cell lines

Cell line source(s)

The HEK293T cell line was from the Cytogenetics and Cell Authentication Core at MD Anderson Cancer Center. The human liver cell lines, Hep3B, HepG2 (ATCC), Mahlavu, PLC/PRF/5(ATCC), HA59T, Tong, and Huh-7, were gifts from Mien-Chie Hung (MD Anderson Cancer Center). The HT1080 cell line (ATCC) was from Boyi Gan's lab stock (MD Anderson Cancer Center). The immortalized human hepatocyte cell line MIHA was purchased from Albert Einstein College of Medicine. Two immortalized mouse liver progenitor cell lines, PHM (p53-null and c-Myc-overexpressing) and PHR (p53-null and H-RasV12-overexpressing), were from Lars Zender (University of Tübingen) and Wen Xue (University of Massachusetts Medical School), respectively.

Authentication

Short tandem repeat (STR) profiling was done by ATCC and MD Anderson’s Characterized Cell Line Core Facility.

Mycoplasma contamination

All cell lines were confirmed to be mycoplasma free with a mycoplasma detection kit and treated with Plasmocin for the Prevention of mycoplasma contamination.

Commonly misidentified lines
(See ICLAC register)

None.

Animals and other organisms

Policy information about studies involving animals; ARRIVE guidelines recommended for reporting animal research

Laboratory animals

Species: mouse

Gender: male

Strains:
NSG mice, MD Anderson’s internal supply
Cre-ERT2 mice (C57BL/6), The Jackson Laboratory Stock# 008085; RRID: IMSR_JAX:008085
Albumin-Cre mice (C57/B6), The Jackson Laboratory Stock# 003574; RRID: IMSR_JAX:003574
Lifr conditional knockout mice (with the LoxP-flanked Lifr allele, C57BL/6), generated in this study

Ages and procedures: (1) PDX model: 6 weeks old at the time of PDX tumor implantation; (2) DEN-induced liver cancer model: 14 days old at the time of DEN injection; (3) Sleeping beauty transposon-mediated oncogene-induced liver cancer model: 8-12 weeks old at the time of hydrodynamic tail vein injection of plasmids.

Mice were housed at 70F-74F (set point: 72F) with 40%-55% humidity (set point: 45%). The light cycle of animal rooms is 12 h of light and 12 h of dark.

Wild animals

No wild animals were used.

Field-collected samples

No field-collected samples were used.

Ethics oversight

All animal studies were performed in accordance with a protocol approved by the Institutional Animal Care and Use Committee of MD Anderson Cancer Center.

Note that full information on the approval of the study protocol must also be provided in the manuscript.

Human research participants

Policy information about studies involving human research participants

Population characteristics

Our research did not involve human subjects, but used human samples from the Xie laboratory at the Shanghai Institute of Nutrition and Health. All HCC tissues and paired adjacent tissues were collected with written informed consent from Eastern Hepatobiliary Surgery Hospital, Second Military Medical University (Shanghai, China) between 2013 and 2015. They were shared with us as de-identified specimens.

Recruitment

We did not recruit any patients.

Ethics oversight | The collection and use of human samples were approved by the Ethics Committee of Shanghai Institutes for Biological Sciences, Chinese Academy of Sciences (Shanghai, China) following the declaration of Helsinki ethical guidelines.

Note that full information on the approval of the study protocol must also be provided in the manuscript.

Flow Cytometry

Plots

Confirm that:

☒ The axis labels state the marker and fluorochrome used (e.g. CD4-FITC).

☒ The axis scales are clearly visible. Include numbers along axes only for bottom left plot of group (a 'group' is an analysis of identical markers).

☒ All plots are contour plots with outliers or pseudocolor plots.

☒ A numerical value for number of cells or percentage (with statistics) is provided.

Methodology

Sample preparation	For cultured cell lines, cells were incubated with the Accutase Cell Detachment Solution (BioLegend, 423201) and were washed twice with PBS. 1 × 10^6 cells per sample were used for staining.
Instrument	Cells were processed on an Invitrogen Attune NxT Acoustic Focusing Cytometer.
Software	Cells were processed on an Invitrogen Attune NxT Acoustic Focusing Cytometer and analyzed by FlowJo software (FlowJo, LLC, version 10.4).
Cell population abundance	At least 5,000 cells were analyzed for each sample.
Gating strategy	Gating strategies are provided in the Supplementary Information.

☒ Tick this box to confirm that a figure exemplifying the gating strategy is provided in the Supplementary Information.
